# Predicting Climate Change Impacts on the Amount and Duration of Autumn Colors in a New England Forest

**DOI:** 10.1371/journal.pone.0057373

**Published:** 2013-03-08

**Authors:** Marco Archetti, Andrew D. Richardson, John O'Keefe, Nicolas Delpierre

**Affiliations:** 1 Department of Organismic and Evolutionary Biology, Harvard University, Cambridge, Massachusetts, United States of America; 2 Harvard Forest, Petersham, Massachusetts, United States of America; 3 Laboratoire Ecologie Systématique et Evolution, Université Paris-Sud, Orsay, France; DOE Pacific Northwest National Laboratory, United States of America

## Abstract

Climate change affects the phenology of many species. As temperature and precipitation are thought to control autumn color change in temperate deciduous trees, it is possible that climate change might also affect the phenology of autumn colors. Using long-term data for eight tree species in a New England hardwood forest, we show that the timing and cumulative amount of autumn color are correlated with variation in temperature and precipitation at specific times of the year. A phenological model driven by accumulated cold degree-days and photoperiod reproduces most of the interspecific and interannual variability in the timing of autumn colors. We use this process-oriented model to predict changes in the phenology of autumn colors to 2099, showing that, while responses vary among species, climate change under standard IPCC projections will lead to an overall increase in the amount of autumn colors for most species.

## Introduction

### Climate change and autumn colors

Temperature affects biological processes ranging from the molecular to the ecological level. It is not surprising, therefore, that climate change is altering the phenology of many species [1]–[7]. In plants, the impacts of climate change on spring phenology (flowering) are well documented [8]–[13]. Much less is known, however, about how warming temperatures and altered precipitation regimes affect autumn phenology, specifically as related to leaf coloration and senescence.

About 15% of the tree species of the temperate regions of the world change their leaf color from green to yellow or red in autumn, a percentage that can reach 70% in some regions like New England (Northeast USA) [14]–[15]. As leaf color change and leaf fall are thought to be controlled by temperature and precipitation [16]–[18], it is possible that climate change may also affect autumn phenology, with obvious biological and ecological implications [19].

At the continental scale, warmer autumns have for instance been related to lower net carbon fixation [20]–[21], as a consequence of a higher enhancement of ecosystem respiration than the concomitant enhancement of gross photosynthesis. At a local scale, temperate deciduous forests may on the contrary show a higher annual net carbon fixation during warmer autumn as a consequence of an extended leafy season [22]. There is further evidence that the asynchrony of autumn phenology may alter the competition between co-occurring plant species, either in the case of symmetric (between understory plants - all plants being light-limited by the overstorey canopy) [23] or asymmetric (between overstory and understory plants) [24] competition.

Additionally, the potential impact of climate change on the intensity and duration of autumn coloration is, in some regions, of enormous economic importance [25]. Autumn tourism—much of which is to participate in so-called ‘leaf peeping’—contributes billions of dollars each year to the economies of the states of the eastern U.S.A. and provinces in adjacent Canada. If climate change reduces the duration of autumn color display, or results in less vibrant displays, future tourism revenues will likely be reduced.

### Rationale of the study

In order to predict how autumn colors may respond to forecast changes in environmental drivers, we analyzed data on leaf color change collected annually between 1993 and 2010 in a New England forest for eight study-species that develop anthocyanins in autumn. For each species we calculated the average percentage of colored leaves and of fallen leaves for each day of the year for the 18 years during which the data were gathered. We investigated correlations between temperature and precipitation during different times of the year, and the timing of various autumn color thresholds and leaf fall dates. We compared two types of models to explain autumn coloration and leaf fall. First, we used an empirical approach [26] based on stepwise multiple linear regression, with monthly means of temperature and precipitation as the candidate independent variables. Second, we used a more mechanistic approach using a cold-degree-day photoperiod-dependent model [27]. The correlation analysis and empirical modeling allow us to identify environmental drivers that may be missing from the mechanistic model, which is highly constrained in its structure, and which does not, for example, account for relationships between precipitation and autumn color. We evaluated the models against the observational data using cross-validation methods. We then used the most robust modeling approach, in conjunction with IPCC climate projections, to forecast changes in the phenology of autumn color and leaf fall, between now and the year 2099.

## Materials and Methods

### Data

We analyzed data on the autumn phenology of *Acer rubrum* (red maple), *Acer saccharum* (sugar maple), *Fraxinus americana* (white ash), *Nyssa sylvatica* (black gum), *Prunus serotina* (black cherry), *Quercus alba* (white oak), *Quercus rubra* (red oak) and *Quercus velutina* (black oak) at Harvard Forest, a research area owned and managed by Harvard University, in Petersham, Massachusetts, USA (Prospect Hill Tract; 42.54 °N, 72.18 °W). For more than twenty years, phenological observations have been made, every 3–7 days in spring and autumn [18], [28], by the same observer. The observed trees (3 to 5 permanently-tagged individuals per species) are located within 1.5 km of the Harvard Forest headquarters at elevations between 335 and 365 m above sea level. The field protocol for autumn observations was finalized in 1993 and here we use observations through the end of 2010. Beginning in September, and continuing through the end of leaf fall, leaf coloration (the percentage of leaves that have changed color on a given tree) and leaf fall (the percentage of leaves that have fallen from a given tree) are estimated for each individual observed. The raw data are available at http://harvardforest.fas.harvard.edu/data/archive.html (datasets HF000, HF001, HF003); the transformed data and the codes used for the analysis are available from the authors, while the final data are in Table S1.

### Measures of autumn color

We used the original data to infer the day (*c*_x_) on which the percentage of colored leaves is *x* and the day (*f*_x_) in which the percentage of fallen leaves is *x* (where *x* may take a value of 10, 25, 50, 75 or 90 percent). Assuming that both color and leaf retention change as a linear function between the days in which the observations were recorded, we derived *c*_x_ using the formula
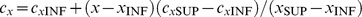



where *x*_INF_ and *x*_SUP_ are the available measure immediately lower and higher than *x*; *f*_x_ was derived in a similar way as
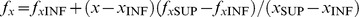



For a few species, in some years (18 in a total of 2304 data points, that is 0.65% of the data), certain thresholds (mainly *c*_10_ and *c*_25_) had already been reached before the first field observations were made: in these cases, rather than extrapolate backwards, we simply treated these as missing data.

**Figure 1:**
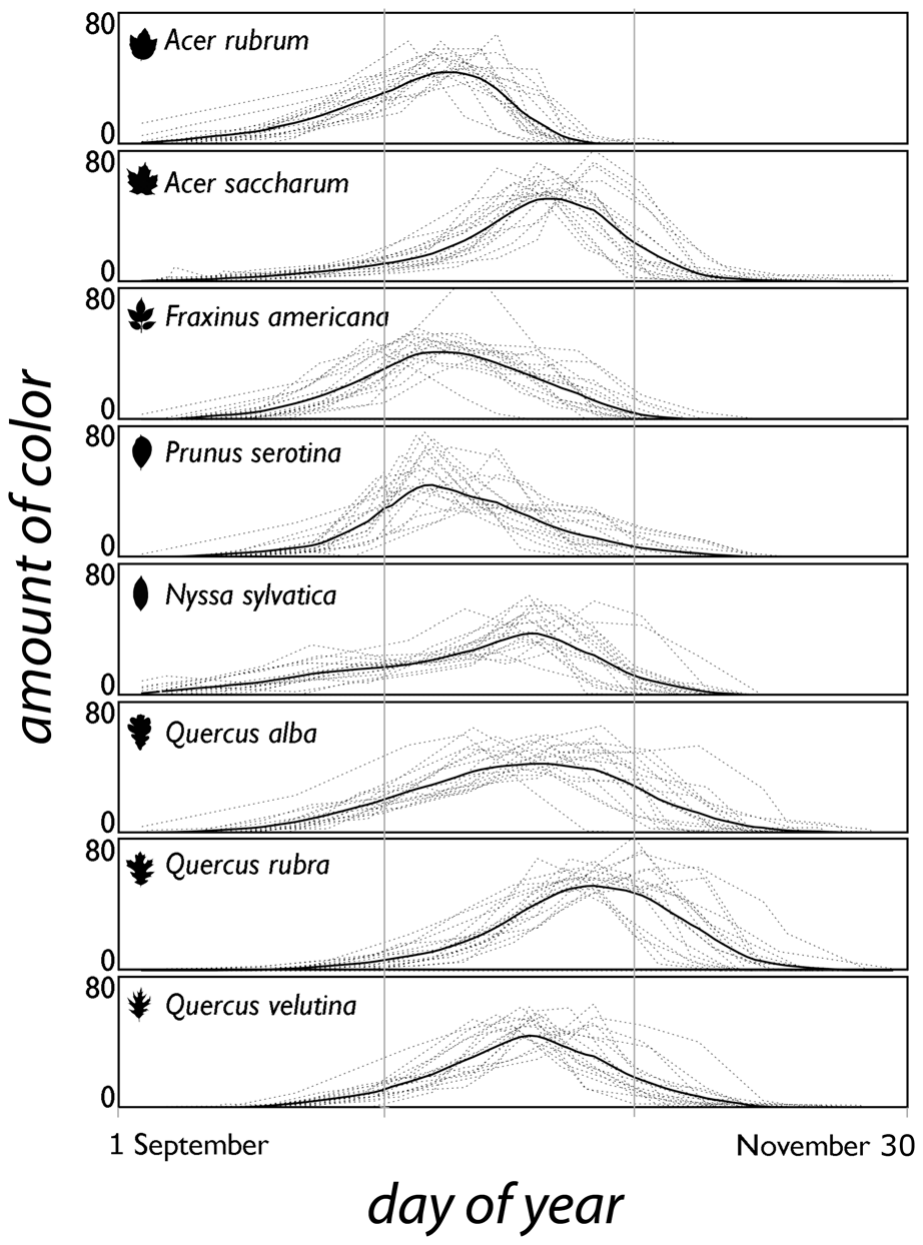
The amount of autumn colors over time for eight deciduous broadleaf species that turn red in autumn. The amount of autumn color (0–100) is calculated as *i*_n_(100−*j*_n_) on day *n*, where the percentage of red leaves *i*_n_ is multiplied by the percentage of leaves retained (100−*j*_n_). Individual years (1993–2010) are shown by dotted lines, and their average by the thick curve.

We also used *c*_x_ and *f*_x_ to build two different measures of abundance of autumn color: *d*_x_ = *f*_90_−*c*_x_ measures the *duration* of autumn color as the number of days between the day when a percentage *x* of the leaves are red (*c*_x_) and the day when 90% of the leaves have fallen (*f*_90_). The *amount* of autumn color is measured by (*i*_n_−*i*_n−1_)*y*_n−1_+(*i*_n_−*i*_n−1_)(*y*_n_−*y*_n−1_)/2 if *y*_n_>*y*_n−1_ and by (*i*_n_−*i*_n−1_)*y*_n_+(*i*_n_−*i*_n−1_)(*y*_n−1_−*y*_n_)/2 if *y*_n_<*y*_n−1_, where *y*_n_ = *r*_n_(1−*t*_n_/100); *r*_n_ is the percentage of red leaves, *t*_n_ is the percentage of leaves retained, *i*_n_ is the (julian) day when the *n* ^th^ measure (of a total of *m* measures) was taken. The *yearly amount* of autumn color
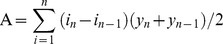



therefore is (in a Cartesian plane), the area below the lines that connect the daily amount of autumn color (see Figure 1). 100 units of *A* correspond to one calendar day in which all leaves are retained and red.

### Correlation analysis and regression modeling

Air temperature and precipitation are measured (daily) at the Harvard Forest near to the trees on which phenological observations have been conducted. Data for the Shaler (1964–2002) and Fisher (2001-present) meteorological stations are available online at the web address given above; any missing observations were filled using measurements from the Harvard Forest EMS AmeriFlux tower, approximately 1 km distant.

For both temperature and precipitation, we first calculated averages (of the daily measures) over all the 1- to 52-week timeframes preceding each day of the year. We then calculated the correlation coefficients between these averages and each of the measures of autumn color (*A*; *c*_x_, *f*_x_, *d*_x_; see above) for each species. Based on the correlation analysis, we identified the periods of the year during which the largest positive and the largest negative correlations were observed with the measures of autumn color.

For our empirical modeling of leaf color threshold dates (*c*_x_) and leaf fall threshold dates (*f*_x_), we calculated monthly means of temperature and precipitation during the leaf-on (May to October) period. We conducted a stepwise multiple linear regression procedure with the monthly mean drivers as candidate independent variables (6 months×2 drivers = 12 candidate variables). We specifically chose a monthly time interval (rather than weekly) for averaging, and restricted our analysis to the leaf-on period, so as to avoid having too many candidate variables, which could increase the likelihood of type 1 (false positive) errors and potentially lead to the inclusion of spuriously correlated variables in the regression. At each iteration of the stepwise procedure, variables that would be significant at a *p*-value of ≤ 0.20 were added to the regression but were subsequently removed if, after other variables were accounted for, the *p*-value exceeded 0.05. We fit a separate model to each *c*_x_ threshold and each *f*_x_ threshold; *A* and *d*_x_ were then calculated from *c*_x_ and *f*_x_. Below, we refer to this Multiple Linear Regressions approach as the MLR model.

### Process-oriented modeling

We used a cold-degree-day photoperiod-dependent (*CDD/P*) model [27]. This model was initially designed to simulate a coloring stage and was further applied in this study to the simulation of a fall stage. Whatever the senescence stage (*c*_x_ or *f*_x_) considered, it is defined in the model by *S_sen_* (arbitrary units) for each day (*doy*) following *D_start_* (the date at which a critical photoperiod *P_start_* is reached), representing the progress of the simulated process. Leaf coloring or fall reaches a given stage (*c*_x_ or *f*_x_) when *S_sen_* reaches a threshold value (*Y_crit_*, arbitrary units). In this model, the time derivative of the state of senescence (*R_sen_*, arbitrary units) on a daily basis is formulated as:
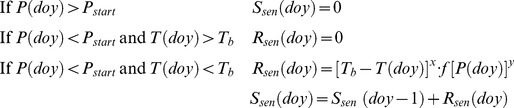



Where *P*(*doy*) is the photoperiod expressed in hours on the day of year *doy*; *T*(*doy*), the daily mean temperature (°C); *T_b_*, the maximum temperature at which the considered senescence (i.e. coloration or fall) process is effective (°C); *f*[*P*(*doy*)], a photoperiod function that can be expressed as follows :
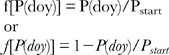



The complete model therefore includes five parameters (*P_start_*, *T_b_*, *x*, *y*, *Y_crit_*). The dummy parameters *x* and *y* may take any of the {0, 1, 2} discrete values, to allow for any absent/proportional/more than proportional effects of temperature and photoperiod to be included. A feature of this model structure is that, depending on the value of *x*, the modeled phenophase can be considered as dependent (*x*>0) or independent (*x* = 0) on cold-degree days. In the latter case, the occurrence of the phenophase is only determined by a threshold photoperiod.

The optimization procedure consisted of exploring the whole space of parameters for *P_start_* (from 10 to 16 h with a 0.5 h step), *T_b_* (from +7 to +30°C with a 0.5°C step), *x*, and *y*. The *Y_crit_* parameter was identified through the Powell (gradient descent) optimization method [29]. Parameter optimization was based on minimizing the model-data mismatch, quantified in terms of root mean squared error.

As with the MLR approach, the CDD/P model was fit independently on leaf color (*c*_x_) and leaf fall (*f*_x_) data for each species. Yet, while the MLR approach was fit on each color and fall stage (e.g. 5 fits for color from *c*_10_ to *c*_90_), we fit the CDD/P model over the complete phenological trajectory (e.g. simultaneously for all five stages from *c*_10_ to *c*_90_ for leaf coloration) defining for each model structure a set of five *Y_crit_* parameters, one per observed stage. We thereafter used the two CDD/P models fit independently on coloring and fall data to predict canopy duration (*d*_x_) and the amount of color (*A*). Statistics were computed using MATLAB version 7.10 (The MathWorks Inc., 2010).

### Robustness assessment of the modeling approaches

The accumulation of a large phenological dataset requires sustained effort over many years, which is why multi-decadal records are relatively scarce. With 18 years worth of data, the Harvard Forest dataset is one of the longest autumn datasets published [28]. However, it is certainly possible that either the statistical (MLR) or process-oriented (CDD/P) approaches could result in models being over-fit to what is still a relatively short time series.

After performing a first fit of both approaches on the full dataset, we evaluated the robustness of each model (i.e. the ability of the model to predict an unknown dataset) by using cross-validation analysis [30], [31]. This approach is commonly used when wholly independent data (e.g., from another site) are unavailable for model testing (for examples in the phenology literature, see [26]). Specifically, we used a one-out cross-validation, which is particularly appropriate when the dataset is relatively small. To conduct the cross-validation, the models were fit sequentially on 17 of 18 points (i.e. years) from the original dataset (‘calibration’) and tested for their ability to simulate the remaining point (‘validation’). This was repeated 18 times, so that each data point was included in the validation set exactly once. Model performance statistics (root mean square error, RMSE, and model efficiency, ME [32]) were then calculated across the 18 validation points.

We assessed the ability of each of the two modeling approaches to maximize the trade-off between model parsimony and goodness-of-fit using Akaike's information criterion, corrected for small samples (AICc [33]).

### Future Climate Scenarios

We used our models to generate forecasts of future shifts in autumn color phenology at Harvard Forest. Thus the model structure is a hypothesis, and the resulting predictions can be tested as future data become available. We ran the models forward using climate projections (2010–2099) for the Harvard Forest grid cell. These were previously generated by Hayhoe et al. [34] using the NOAA GFDL CM2 global coupled climate model [35], statistically downscaled to one-eighth degree (∼10 km) spatial resolution at a daily time step. The CM2 model was run using two scenarios of CO_2_ and other greenhouse gas emissions (the IPCC Special Report on Emission Scenarios [SRES] higher [A1fi] and lower [B1] scenarios [36]). Compared to a 1960–1990 baseline of 7.1°C mean annual temperature and 1100 mm annual precipitation, corresponding values (mean 2070–2099) are 12.0°C and 1270 mm for the A1fi scenario and 9.5°C and 1240 mm for the B1 scenario. Under the A1fi scenario, summer temperature are projected to increase more than temperatures during the rest of the year, while relatively more precipitation will fall during the autumn and winter months, and less during the spring and summer months. Under the B1 scenario, changes in seasonality are negligible, with changes in temperature and precipitation being relatively similar across the year.

## Results

### Variations in phenology

In the 18 years in which the data were collected, autumn color display typically started at the beginning of September, peaked at variable times in October, and lasted until November, with marked differences among species and, within each species, among years (Figure 1; Table S1). Peak color was earliest for *Prunus serotina*, *Acer rubrum* and *Fraxinus americana*, and latest for *Acer saccharum* and the various *Quercus* spp.

**Figure 2:**
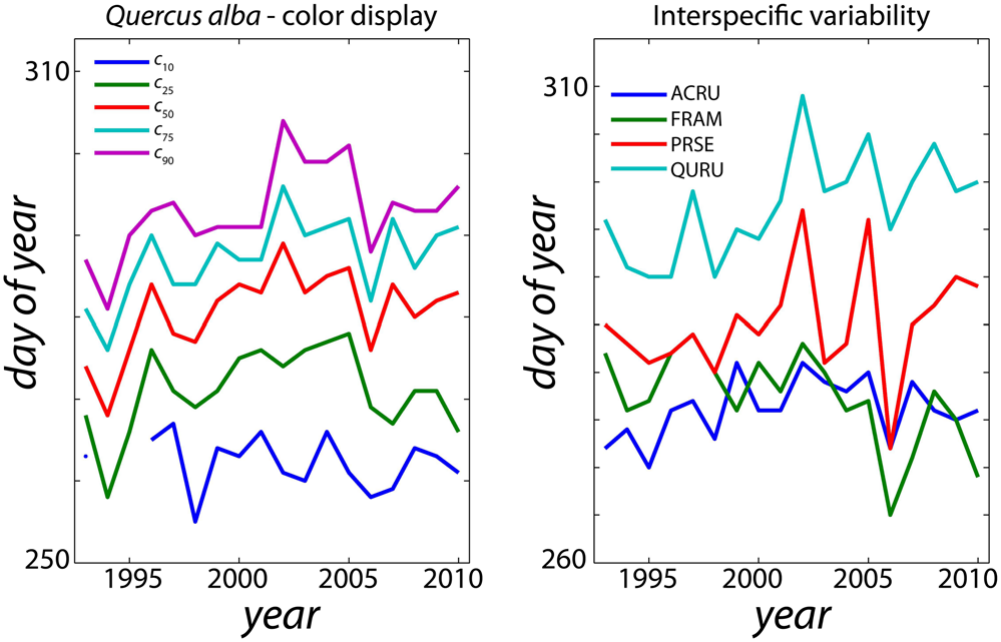
Interannual variability of autumn senescence stages. **2a:** timing of leaf coloration stages (c_10_ = 10% of leaves colored … c_90_ = 90% of leaves coloured) for *Quercus alba*, white oak. **2b:** timing of 50% leaf fall for four species (ACRU  =  *Acer rubrum*; FRAM  =  *Fraxinus americana*, PRSE  =  *Prunus serotina*; QURU  =  *Quercus rubra*).

Year-to-year shifts in the entire sequence of stages are easily seen, with 1994 being a year of early coloration and 2002 being a year of late coloration (example of *Quercus alba*, Figure 2a). The interspecific variability of autumn stages is illustrated with the example of 50% leaf fall, which occurs on average 23 days earlier in *Acer rubrum* than in *Quercus rubra* (Figure 2b). The interannual variability of autumn stages varied from species to species, with, for example, a SD of 3.1 days in *Acer rubrum* and 6.6 days in *Quercus alba* for 50% leaf fall.

**Figure 3:**
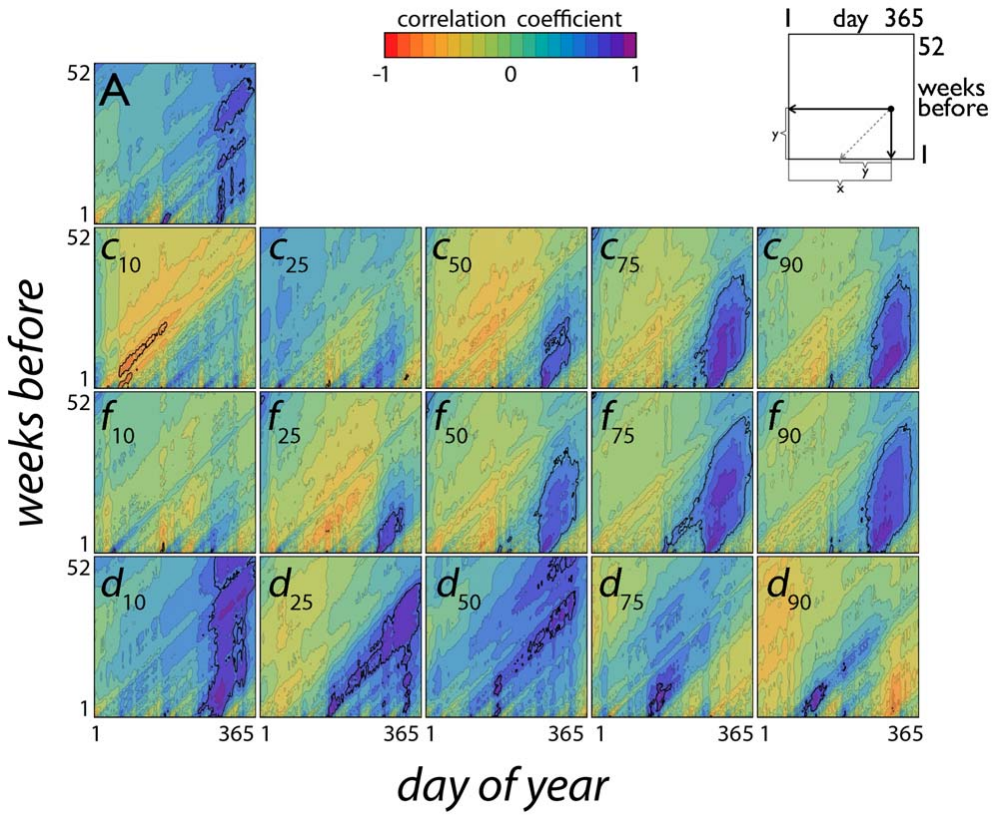
Correlation between interannual variation in temperature and interannual variation in autumn color phenology in red maple, *Acer rubrum*. Each point (*x*,*y*) in each plot represents a time window spanning the *y* weeks (vertical axis) before day *x* (horizontal axis). The color at each point (*x*,*y*) represents the correlation between the average air temperature for the time window (*x*,*y*) and the measure of autumn leaf phenology for that plot: onset of autumn colors (*c*_i_), time of leaf fall (*f*_i_), duration of autumn colors (*d*_i_) and total amount of color (*A*). Values of *R* are shown by colors ranging from orange-red (minimum, negative) to blue-purple (maximum, positive); absolute values of *R*>0.468 (the critical value of the Pearson product-moment correlation coefficient; *p* = 0.05; *d.f.* = 16) are inside the bold lines. Here, both leaf fall and the display of red leaves were shifted significantly later in years with warmer autumn temperatures. Dates of the full display of autumn colors (*c*_75_, *c*_90_) were positively correlated with temperatures from spring through (especially) autumn, while warmer spring temperatures are correlated with earlier onset of color (*c*_10_). Both the duration of autumn colors (*d*_x_) and the total amount of autumn color (*A*) tended to increase in years with warmer temperatures.

The interannual variation of autumn phenology of each species was correlated with interannual variation in temperature and precipitation at specific times of the year. Consider, for example, *Acer rubrum* (Figure 3). Both leaf fall (*f*_x_) and the display of red leaves (*c*_x_) were shifted significantly later in years with warmer autumn temperatures. Dates of the full display of autumn colors (*c*_75_, *c*_90_) were positively correlated with temperatures from spring through autumn (although spring temperature correlations were weaker than those in autumn), but earlier onset of color (*c*_10_) occurred in years with warmer spring temperatures. Both the duration of autumn colors (*d*_x_) and the total amount of autumn color (*A*) tended to increase in years with warmer temperatures, particularly warmer spring and autumn temperatures.

For each species there is a different ‘fingerprint’ to correlations between autumn colors and temperature/precipitation at different times of the year (Figure S1). In *Acer saccharum*, *Nyssa sylvatica*, and *Prunus serotina*, the onset of color and leaf fall were correlated with temperature in a manner that was similar to *Acer rubrum*. In *Fraxinus americana,* advances in the onset of autumn color (*c*_10_), and delays in the full display of autumn color (*c*_90_) occurred in years with warmer temperatures, while leaf fall dates were advanced in years with warmer temperatures. As a consequence, the duration of the full display of autumn color (*d*_90_) was reduced in years with warmer autumn temperatures. In *Quercus velutina,* delays in both leaf coloration and leaf fall were correlated with warmer autumn temperatures, and the total amount of autumn color (A) was positively correlated with summer and autumn temperatures.

Our analysis suggests, therefore, that over the course of the year, interannual variation in temperature is correlated with species-specific and phenophase-specific variation in autumn phenology. Similar patterns are seen when the same analysis is conducted for precipitation (Figure S2). To the extent that these may represent causal relationships, it is therefore quite likely that the autumn phenology of each species will respond to future climate change in a slightly different manner.

### Stepwise regression analysis

In order to increase our understanding of the statistical dependence between autumn phenology and the climate drivers, we conducted a total of 40 stepwise regressions (5 thresholds×8 species) for each of *c*_x_ and *f*_x_ (Table S2). Across all *c*_x_, the mean (±1 SD) *R* ^2^ was 0.49±0.28; for *f*_x_, the corresponding value was 0.44±0.26. However, for 7 of the *c*_x_ regressions, and 6 of the *f*_x_ regressions, no variables were selected by the stepwise procedure, and hence these models had *R* ^2^ = 0.

Mean September temperature was included in 23 of the *c*_x_ regressions, and 27 of the *f*_x_ regressions. In all cases, the regression coefficients were positive, indicating that warmer September temperatures were associated with delayed coloring and leaf fall. By comparison, mean October temperature was included in only 5 of the *c*_x_ regressions and 3 of the *f*_x_ regressions, and the signs of the regression coefficients varied among species.

Temperatures earlier in the growing season were, in some cases, included in the regressions. For example, mean May temperature was included in 11 of the *c*_x_ regressions and 6 of the *f*_x_ regressions. In each of these cases, the regression coefficient was negative, indicating that warmer May temperatures were associated with advanced coloring and leaf fall.

Despite the apparent importance of precipitation indicated by the correlation analyses described above, for no month was mean monthly precipitation included in more than three (of 40) *c*_x_ or *f*_x_ regressions.

### Cold-degree-day modeling

Across all *c*_x_, the mean (±1 SD) *R* ^2^ was 0.43±0.20; for *f*_x_, the corresponding value was 0.34±0.22. Presumably because of its lower degree of flexibility, the CDD/P model did not fit the observations as well as the more highly parameterized MLR model.

In all but one case, the CDD/P model structure yielding the lowest prediction error included cold-degree-days (i.e. a sum of temperature below a certain temperature threshold) as a driving variable for the simulation of *c*_x_ and *f*_x_ phenology (Table S2). Only for leaf fall in *Fraxinus americana* was this model structure unable to simulate the suite of stages better than the null model (which implicitly assumes that photoperiod was the sole trigger of senescence processes, yielding each year the same prediction date for a given stage). In 10 over 80 coloring and fall cases (Table S2), the selected model structure incorporated an interaction effect of photoperiod and cold-degree-days, meaning that a given departure from the base temperature stimulated senescence processes differently as daylength decreased.

### Comparison of modeling approaches

**Figure 4:**
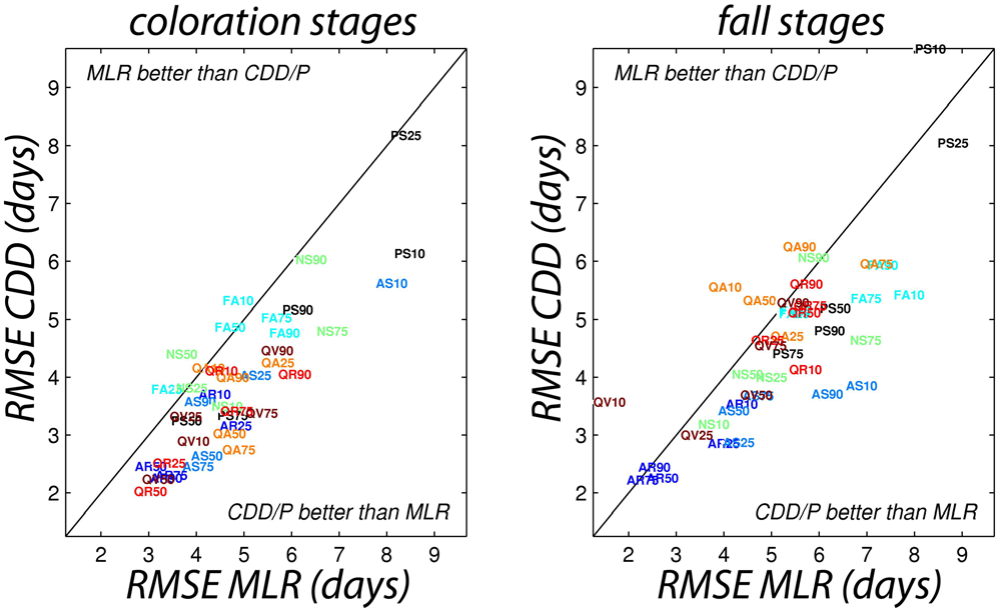
Comparison of the empirical and process-oriented models. Comparison of goodness-of-fit (in terms of RMSE) of empirical (MLR) and process-oriented (CDD/P) models for leaf coloration (left) and leaf fall (right), in a leave-one-out cross-validation analysis. The MLR model is shown to be less robust, as its RMSE is higher (to the right of the 1∶1 line) in a majority of cases.

When fit over the full dataset, the MLR model usually (80% cases) fit the data better (higher modeling efficiency, ME, and lower RMSE) than the CDD/P model (Table 1). In addition, in 69% of cases, the MLR maximised the trade-off between model parsimony and goodness-of-fit: the MLR approach generally resulted in lower Akaike's Information Criterion (AICc) values than the CDD/P approach (Table 1). However, the MLR approach appeared to be somewhat less robust than the CDD/P approach, suggesting that the empirical models may have been over-fit. For example, in the one-out cross-validation analysis, predictions from the CDD/P approach consistently had lower RMSE than those from the MLR approach (Figure 4). This gives us greater confidence in the use of the CDD/P model for forecasting purposes, compared to the MLR approach.

**Table 1:** Empirical (MLR) and process-oriented (CDD/P) model fit statistics, calculated across the entire trajectory of leaf coloration (*c*_10_ … *c*_90_) and leaf fall (*f*_10_ … *f*_90_) for all eight study species.

Phenology	Species	MLR model				CDD model				
		RMSE	ME	P	AICc	RMSE	ME	P	AICc	△AIC
Leaf Color	*Acer rubrum*	2.6	0.93	10	188.7	2.3	0.94	9	162.2	26.6
	*Acer saccharum*	3.3	0.88	14	244.5	3.4	0.88	9	235.7	8.8
	*Fraxinus americana*	2.9	0.93	16	222.3	4.0	0.87	9	260.6	−38.3
	*Nyssa sylvatica*	4.2	0.83	8	270.0	4.0	0.84	9	264.2	5.8
	*Prunus serotina*	4.4	0.91	11	282.5	4.7	0.89	9	289.1	−6.7
	*Quercus alba*	2.4	0.96	16	190.3	3.3	0.92	9	228.5	−38.2
	*Quercus rubra*	2.9	0.92	14	228.1	3.1	0.91	9	224.0	4.1
	*Quercus velutina*	2.6	0.94	17	212.2	3.1	0.91	9	220.1	−7.8
Leaf Fall	*Acer rubrum*	1.8	0.94	15	141.9	2.4	0.89	9	173.6	−31.8
	*Acer saccharum*	3.4	0.85	11	243.7	2.9	0.89	9	207.2	36.6
	*Fraxinus americana*	4.7	0.76	8	295.8	5.1	0.72	9	310.6	−14.8
	*Nyssa sylvatica*	3.2	0.90	17	249.8	4.2	0.83	9	272.5	−22.7
	*Prunus serotina*	5.8	0.78	8	335.3	6.0	0.76	9	342.2	−6.9
	*Quercus alba*	3.5	0.91	13	258.4	4.9	0.84	9	302.9	−44.6
	*Quercus rubra*	4.1	0.80	9	274.9	4.2	0.79	9	278.5	−3.6
	*Quercus velutina*	3.0	0.91	14	234.2	3.5	0.87	9	246.4	−12.2

AICc  =  Akaike's Information Criterion, corrected for small samples (△AIC  =  AICc(MLR) – AICc(CDD/P)); ME  =  model efficiency; P  =  number of fit parameters. ACRU: *Acer rubrum*; ACSA: *Acer saccharum*; FRAM: *Fraxins americana*; NYSY: *Nyssa sylvatica*; PRSE: *Prunus serotina*; QUAL: *Quercus alba*; QURU: *Quercus rubra*: QUVE: *Quercus velutina*.

### Phenological Forecasts

**Figure 5:**
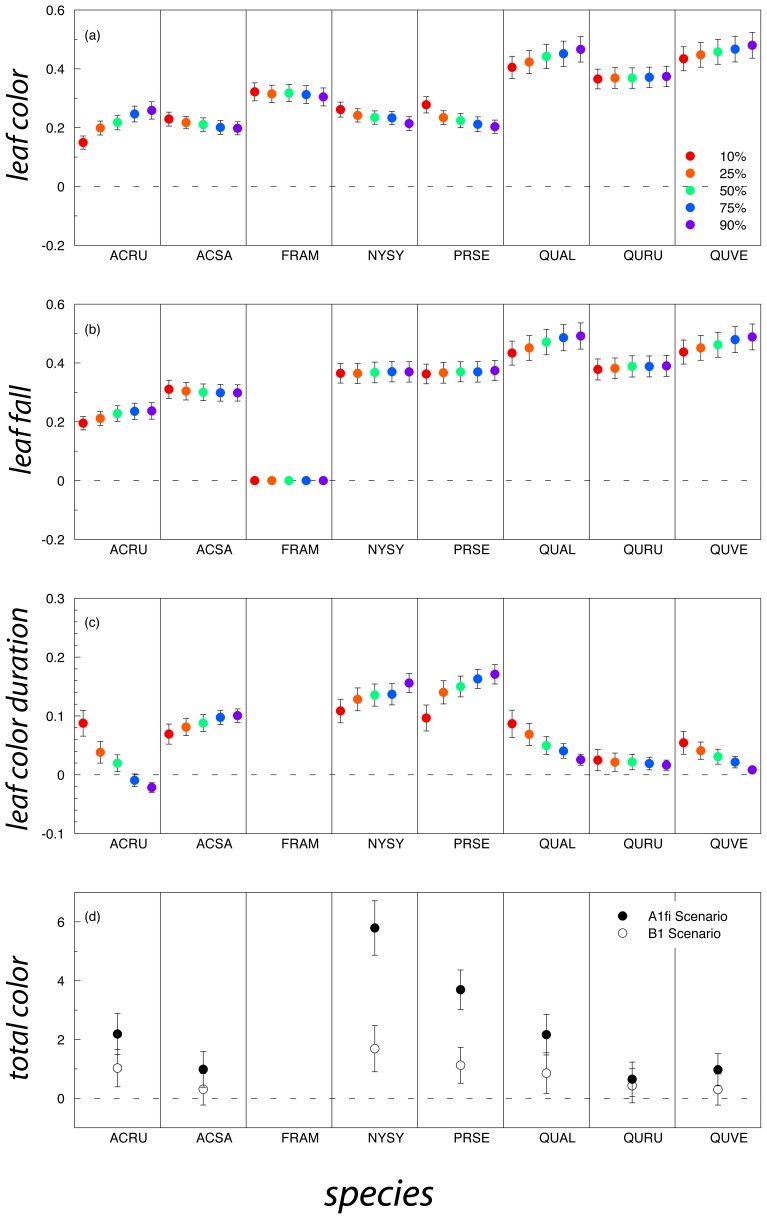
Projected rates of change in the timing of leaf coloration and leaf fall (5a and 5b; dates at which thresholds of 10%, 25%, 50%, 75% and 90% were reached), leaf color duration (5c; number of days between different leaf color duration thresholds and 90% leaf fall), and total amount of autumn colors (5d). For each species, the process-oriented (CDD/P) model, calibrated to 18 years of field data, was run forward using statistically downscaled climate projections from the GFDL CM2 model (IPCC A1fi and B1 scenarios; only A1fi scenario results shown in panels a through c). Projected rates of change (as plotted on the y-axis) were then calculated as the slope of the linear regression line between each phenological variable and year, over the period 2010–2099. Thus, for panels a through c, units are days per year, whereas for d, units are amount of color/year. ACRU: *Acer rubrum*; ACSA: *Acer saccharum*; FRAM: *Fraxinus americana*; NYSY: *Nyssa sylvatica*; PRSE: *Prunus serotina*; QUAL: *Quercus alba*; QURU: *Quercus rubra*: QUVE: *Quercus velutina*.

For modeled future dates of leaf color (*c*_x_) and leaf fall (*f*_x_), we fit a linear regression to estimate the predicted rates of change (days per year) in autumn phenology over the period 2010–2099. We conducted a similar analysis for canopy duration (*d*_x_) and total color (*A*). This was done using the final models identified by both the MLR and CDD/P approaches, keeping in mind that the cross-validation analysis indicated the latter approach to be more robust. Indeed, we found that when run under future climate scenarios, the MLR predictions were sometimes not reliable: ‘crossing-over’ commonly occurred, for some species as early as 2020 or 2030, so that (for example) *f*_50_ was predicted to occur before *f*_25_. These inconsistencies were particularly common for both leaf coloration and leaf fall for two species, *Fraxinus americana* and *Quercus alba*. Of the eight species considered, *Acer rubrum* and *Quercus velutina* were the only species for which crossing-over was not observed to occur. For this reason, we focus our analysis on the forecasts generated with the CDD/P model, acknowledging, however, that (i) this approach may omit important drivers (specifically, precipitation) of autumn leaf phenology and (ii) this approach also predicted dubious patterns in the case of *Fraxinus americana*, for which e.g. *c*_90_ (90% canopy coloration) was predicted to occur after *f*_90_ (90% leaf fall) originating from the inability of the CDD/P model to describe the current interannual variations of leaf fall in this sole species. These results, along with uncertainty estimates (indicating 95% confidence intervals on slope estimates, rather than the uncertainty in phenology model parameters or model structure [37]), are shown in Figure 5.

For the CDD/P approach, a shift towards later occurrences of a given *c*_x_ or *f*_x_ stage is the rule (Figure 5). In some species, such as *Acer rubrum*, *Quercus alba*, and *Quercus velutina*, shifts towards later leaf color (Figure 5a) and leaf fall dates (Figure 5b) are somewhat smaller for earlier thresholds (e.g. *c*_10_, *f*_10_) than later thresholds (e.g. *c*_90_, *f*_90_). For other species, all stages of leaf coloring and leaf fall are predicted to shift by essentially the same amount. Across all thresholds, leaf color duration (Figure 5c) is predicted to increase (by about 0.1 d/y) for *Acer saccharum*, *Nyssa sylvatica*, and *Prunus serotina*, but decrease (by about 0.3 d/y) for *Fraxinus americana*.

The projected change in total amount of color (*A*) is generally positive for all species (Figure 5d). The projected change is substantially larger for the A1fi scenario (higher CO_2_ emissions, larger rise in mean annual temperature and larger increase in annual precipitation) than the B1 scenario (lower CO_2_ emissions, smaller rise in mean annual temperature smaller increase in annual precipitation). Under the B1 scenario, the 95% confidence interval on the slope estimate includes zero for several species. We notice that the CDD/P model (fitted, independently on coloration and fall data) could not predict a consistent trend for *Fraxinus Americana*, for which, for instance, full leaf loss was predicted to occur before full coloration by year 2075. The strongest response to the A1fi scenario is predicted for *Nyssa sylvatica* (+5 units/y), while little or no change in total color is predicted for *Acer saccharum*, a species that is especially popular with leaf peepers. We note that for *Acer rubrum* and *Quercus velutina*, the only two species for which MLR predictions were considered reliable, the responses to the A1fi scenario are much smaller for the CDD/P approach (+2 and +1 units/y, respectively) than the MLR approach (+7 and +9 units/y, respectively).

## Discussion

Our results demonstrate substantial year-to-year variability in the timing and amount of autumn color for the eight species considered. Both the empirical, statistical method (MLR approach, modeling phenological transition dates as a function of monthly precipitation and temperature during the current year's growing season) and the more process-oriented model (CDD/P approach, simulating the influence of cold-degree-days interacting with photoperiod on senescence processes) could be successfully fit to the data, allowing us to reject the null hypothesis that these events are controlled strictly by photoperiod. The CDD/P model was shown, by a one-out cross-validation analysis, to be more robust than the MLR model. The stepwise regression model is wholly empirical, and imposes no formal structure on the relationships between phenological states and meteorological drivers. By comparison, the CDD/P model structure is based on hypotheses [27] about how cold temperatures and/or photoperiod combine to regulate autumn phenology. Furthermore, whereas in the empirical approach the model was estimated separately for each individual phenological threshold, in the CDD/P model the entire progression through all five thresholds (*x* = 10%, 25%, 50%, 75%, 90%) for each of *c*_x_ and *f*_x_ was predicted with a single model

Sensitivity to temperatures at specific times of the year varied among species. For most species, we found that a warm September delayed leaf coloring, and in some cases a warm May advanced coloring. In just a few cases was precipitation in any month included as a statistically significant model driver. Covariation between temperature and precipitation (e.g., warmer Septembers tend to be dry Septembers) may explain why both temperature and precipitation in the same month were rarely included in a single MLR model. Additionally, the monthly averaging used in the regression analysis may have been too coarse, but this approach (e.g. rather than weekly averaging) was selected to minimize the number of candidate independent variables.

Various hypotheses about the environmental controls on autumn coloration and senescence have been proposed [16], but these have not systematically been translated into mechanistic models with good predictive power. Most models developed to date focus on air temperature (sometimes in conjunction with photoperiod) as the primary driver of autumn phenological transitions (e.g. [18], [27], [38]). While empirical analyses, such as performed here (see also the ‘random forest’ decision tree approach [26]), do not provide insight into the underlying mechanisms, they can help us identify the drivers that must be included in a model. We therefore propose that the next generation of mechanistic models of autumn phenology should be structured so as to include interacting functions of temperature and precipitation (or more likely variables related to soil water balance, such as soil moisture or Palmer Drought Index).

Previous modeling studies have generally concluded that autumn leaf coloring and autumn leaf fall in temperate deciduous species will be delayed in the future as continued warming due to climate change occurs. For example, Lebourgeois et al. [26] predict that by 2100, leaf coloring would be delayed, on average, by 13 days compared to the present. Delpierre et al. [27] used a modeling analysis to predict a trend towards delayed leaf coloring of 1.4 and 1.7 days per decade in *Fagus sylvatica* and *Quercus petraea*, respectively, over the 1951–2099 period. Similarly, using the Delpierre et al. 's cold-degree-day model, Vitasse et al. [38] predicted delayed autumn senescence trends (through 2100) of between 1.4 and 2.3 days per decade in the same *Fagus* and *Quercus* species. Our model-based predictions are largely consistent with these estimates (e.g. Figure 5). However, our results further predict that impacts of climate change will likely vary not only among species, but also among specific phenophases—and thus, for example, dates of 10% and 90% leaf color or leaf fall may not shift exactly in parallel. This might help explain previous conflicting suggestions that warmer temperatures may advance or delay leaf coloring [2], [19], [27], [38]–[41]. We put these forward as predictions that should be tested as additional data become available in coming years, or as improved mechanistic models of autumn phenology are developed.

In conclusion, we have shown that forecasting autumn phenology under the IPCC A1fi scenario predicts increases in the amount of autumn color in a New England forest. While the response to changing temperatures and precipitation is species-specific, climate change is expected to have a substantial impact overall on the timing and duration of autumn colors. This may have a dramatic impact on both ecosystem-level C cycling [19] and competitive interactions between species [42], as well as on the landscape and economy of New England and other regions where changes in the timing of autumn leaf colors are one of the most clearly visible indicators of climate change.

## Supporting Information

Figure S1 **Impact of temperature on the phenology of autumn colours and leaf fall.** Each point (*x*,*y*) in each plot represents a time window spanning the *y* weeks (vertical axis) before day *x* (horizontal axis). The color at each point (*x*,*y*) represents the correlation between the average air *temperature* for the time window (*x*,*y*) and the measure of autumn leaf phenology for that plot: onset of autumn colors (*c*_i_), time of leaf fall (*f*_i_), duration of autumn colors (*d*_i_) and total amount of color (*A*). Values of *R* are shown by colors ranging from orange-red (minimum, negative) to blue-purple (maximum, positive); absolute values of *R*>0.468 (the critical value of the Pearson product-moment correlation coefficient; *p* = 0.05; *d.f.* = 16) are inside the bold lines.
(PDF)

Figure S2 **Impact of precipitation on the phenology of autumn colours and leaf fall.** Same as Figure S1 but for *precipitation* rather than *temperature*.
(PDF)

Table S1 **Variables and species.** The IDs and the values of all the variables (*c*_i_, *f*_i_, *d*_i_, *A*) for all years, for the 8 species used in the analysis.
(XLS)

Table S2 **Parameters and statistics of model fits.** Models were fit on the complete dataset. MLR model: P  =  number of parameters estimated in regression model. Temperature and Precipitation columns indicate months that were selected for inclusion in the regression model. + and − signs denote the sign of the regression coefficient. CDD/P model: parameters described in the text. F(P(*doy*)) refers to the use of the first or second function for simulating the interacting effect of photoperiod on the temperature dependence of phenological processes (see text for details).
(DOC)
