# Supplementary material for: Predicting Climate Change Impacts on the Amount and Duration of Autumn Colors in a New England Forest
Source: PLoS One. 2013 Mar 8;8(3):e57373. doi: 10.1371/journal.pone.0057373 (PMC3592872; doi:10.1371/journal.pone.0057373)

Acer rubrum

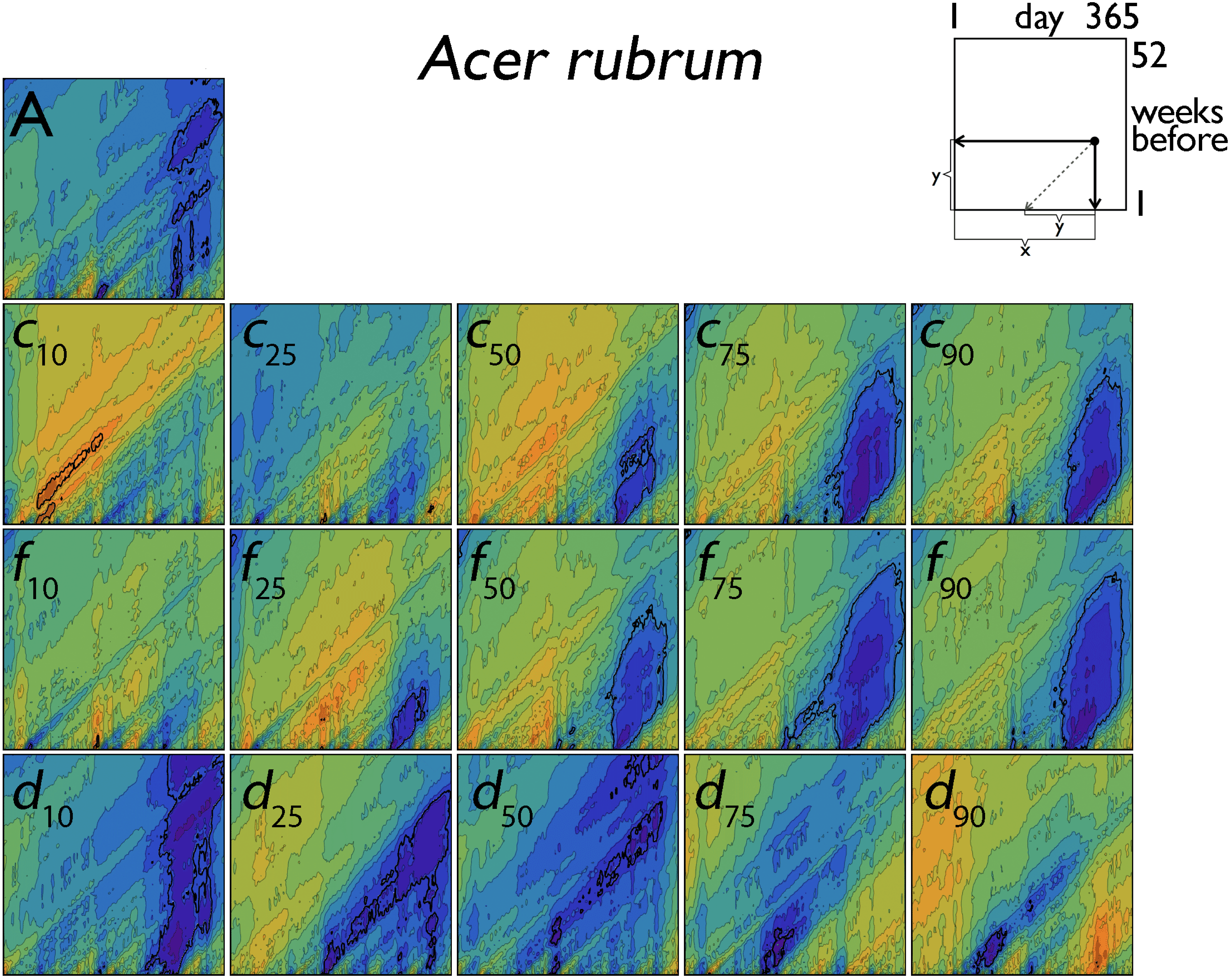

day
1
365
52
weeks before
1
y
y
x
A
c_{10}
c_{25}
c_{50}
c_{75}
c_{90}
f_{10}
f_{25}
f_{50}
f_{75}
f_{90}
d_{10}
d_{25}
d_{50}
d_{75}
d_{90}

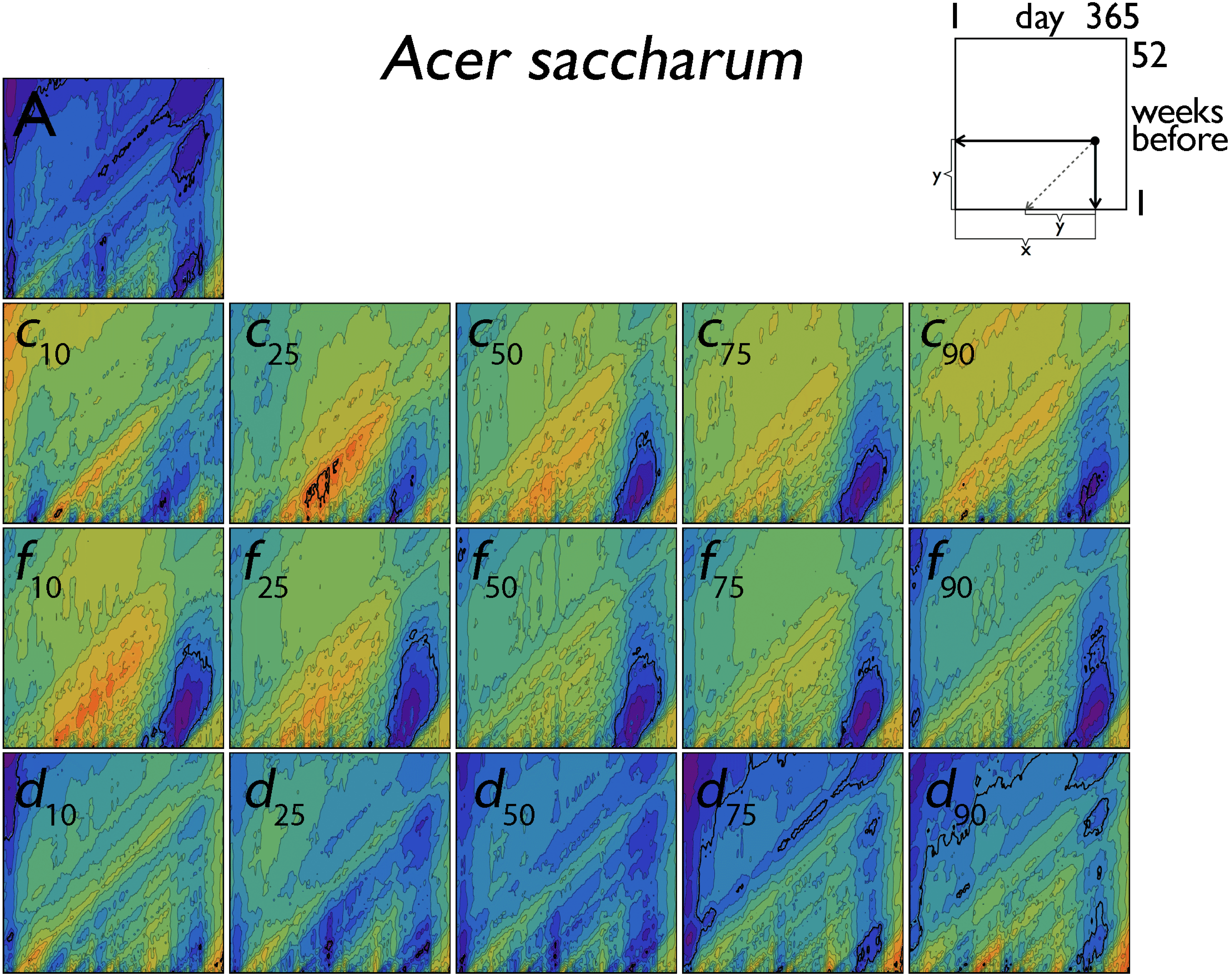

Acer saccharum
1
day
365
52
weeks
before
1
y
y
x
A
c10
c25
c50
c75
c90
f10
f25
f50
f75
f90
d10
d25
d50
d75
d90

Fraxinus americana

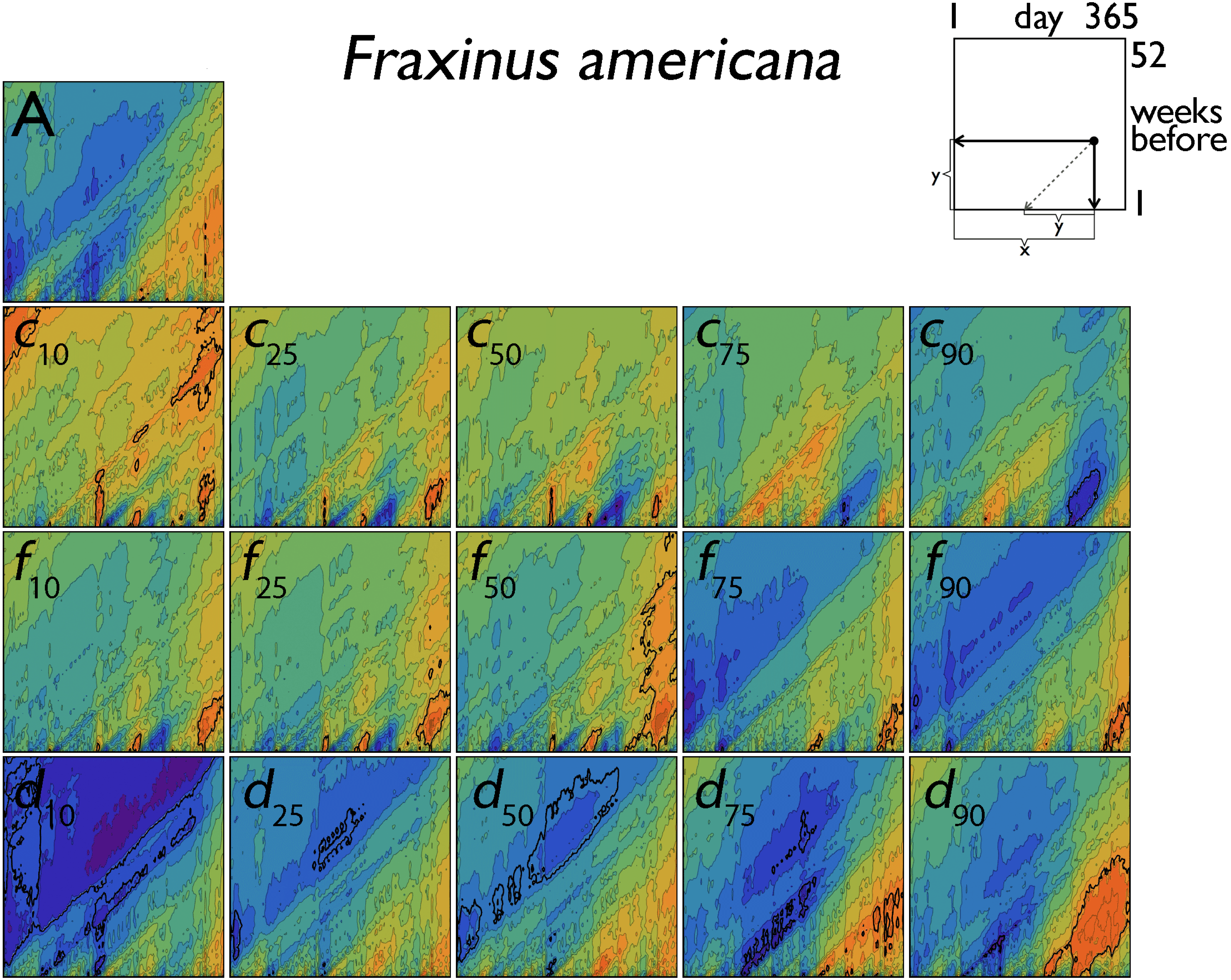

Nyssa sylvatica

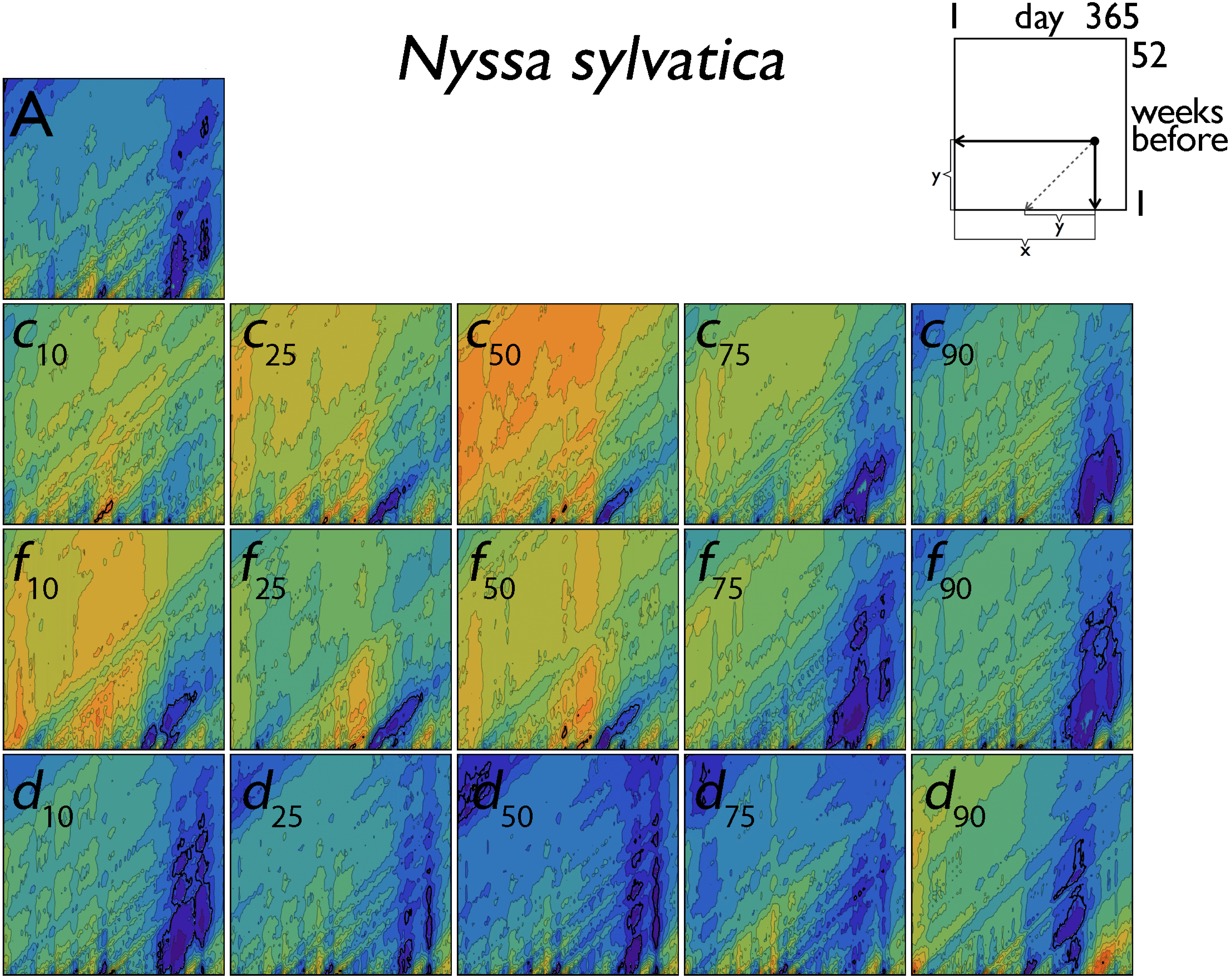

Prunus serotina

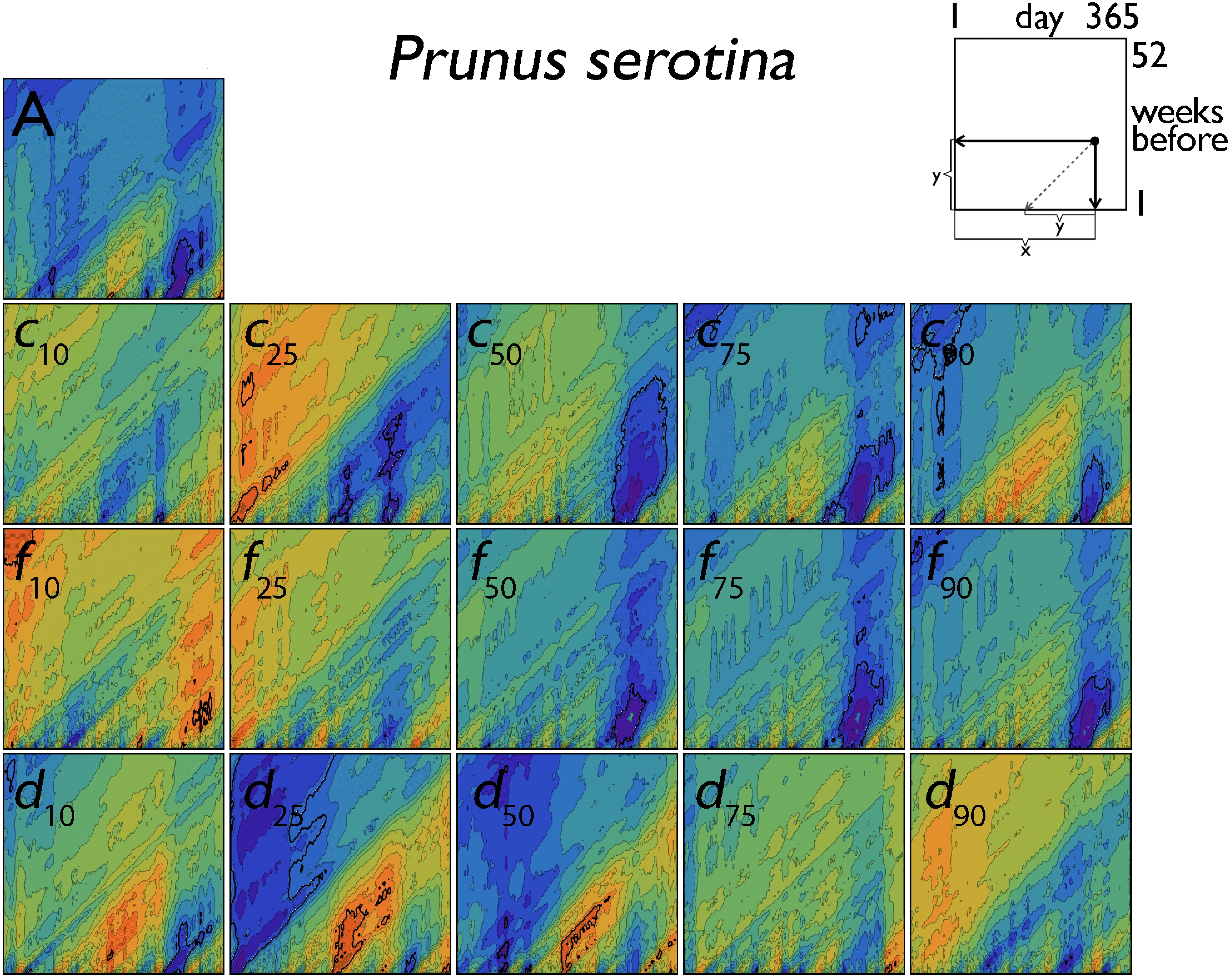

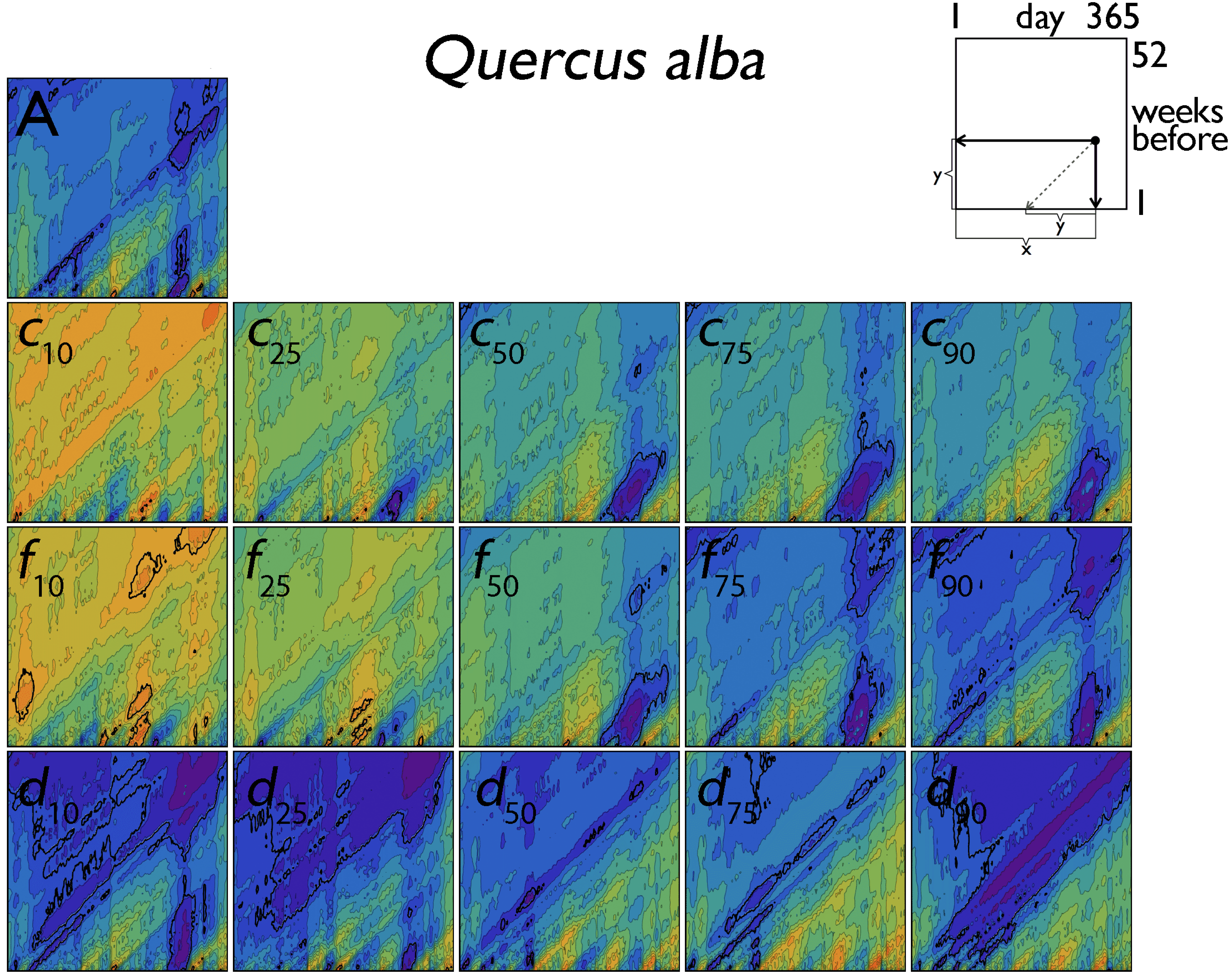
Quercus alba
1
day
365
52
weeks
before
1
y
y
x
A
c10
c25
c50
c75
c90
f10
f25
f50
f75
f90
d10
d25
d50
d75
d90

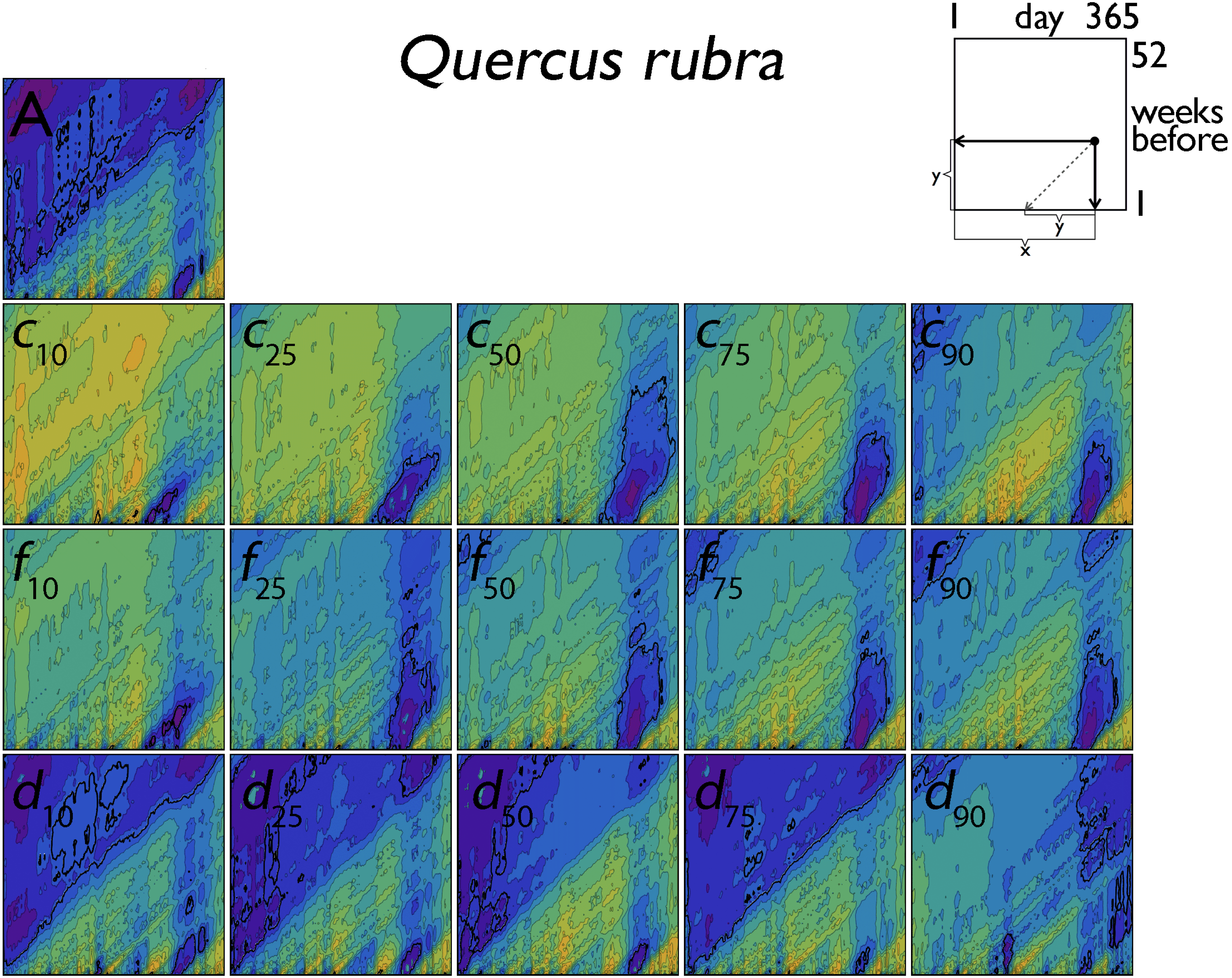

Quercus rubra
1
day
365
52
weeks
before
1
y
y
x
A
c10
c25
c50
c75
c90
f10
f25
f50
f75
f90
d10
d25
d50
d75
d90

Quercus velutina

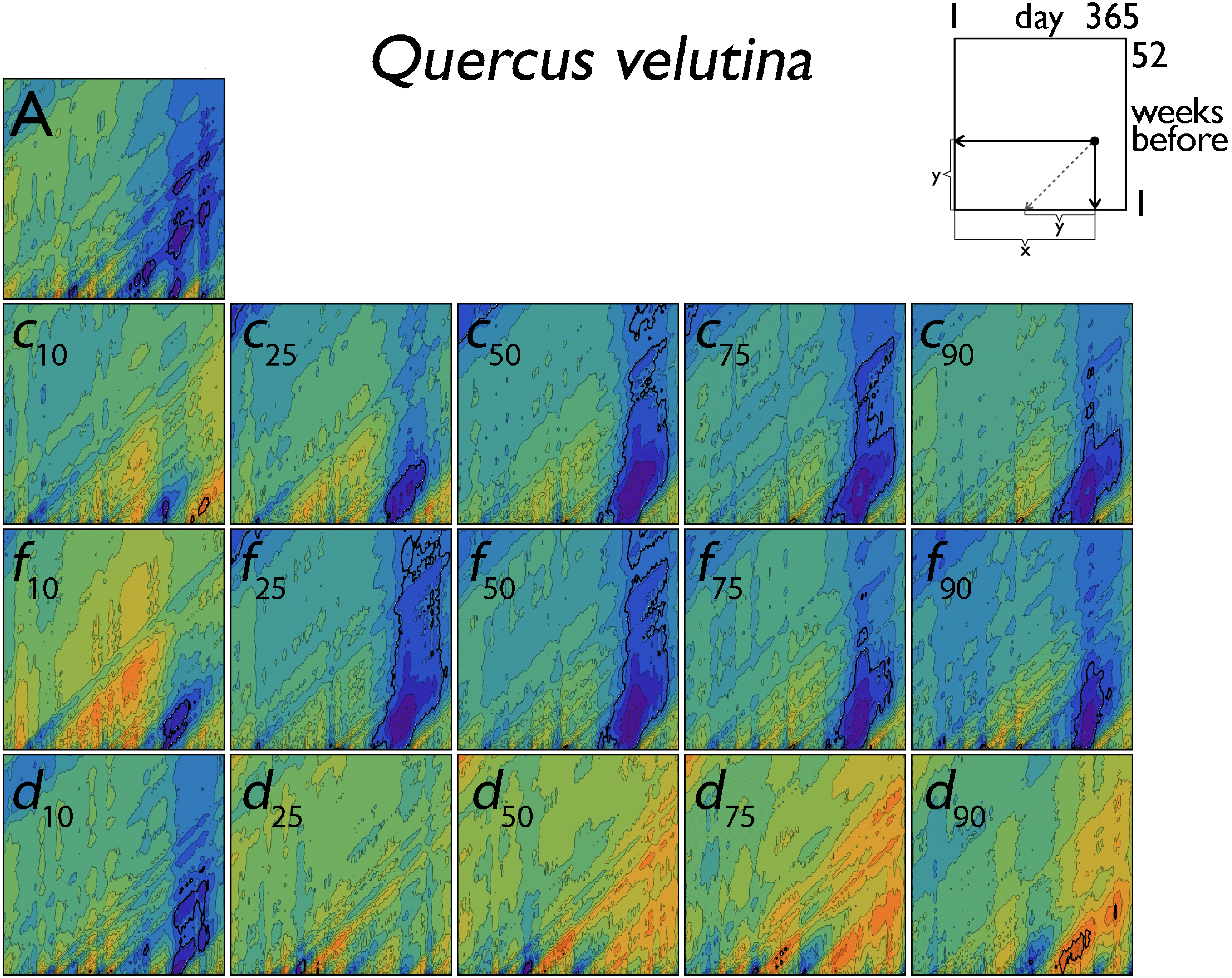

Supplement: Figure S1 — Impact of temperature on the phenology of autumn colours and leaf fall. Each point (x,y) in each plot represents a time window spanning the y weeks (vertical axis) before day x (horizontal axis). The color at each point (x,y) represents the correlation between the average air temperature for the time window (x,y) and the measure of autumn leaf phenology for that plot: onset of autumn colors (c i), time of leaf fall (f i), duration of autumn colors (d i) and total amount of color (A). Values of R are shown by colors ranging from orange-red (minimum, negative) to blue-purple (maximum, positive); absolute values of R>0.468 (the critical value of the Pearson product-moment correlation coefficient; p = 0.05; d.f. = 16) are inside the bold lines. (PDF) [file pone.0057373.s001.pdf]
