# Supplementary material for: Predicting Climate Change Impacts on the Amount and Duration of Autumn Colors in a New England Forest
Source: PLoS One. 2013 Mar 8;8(3):e57373. doi: 10.1371/journal.pone.0057373 (PMC3592872; doi:10.1371/journal.pone.0057373)

Acer rubrum

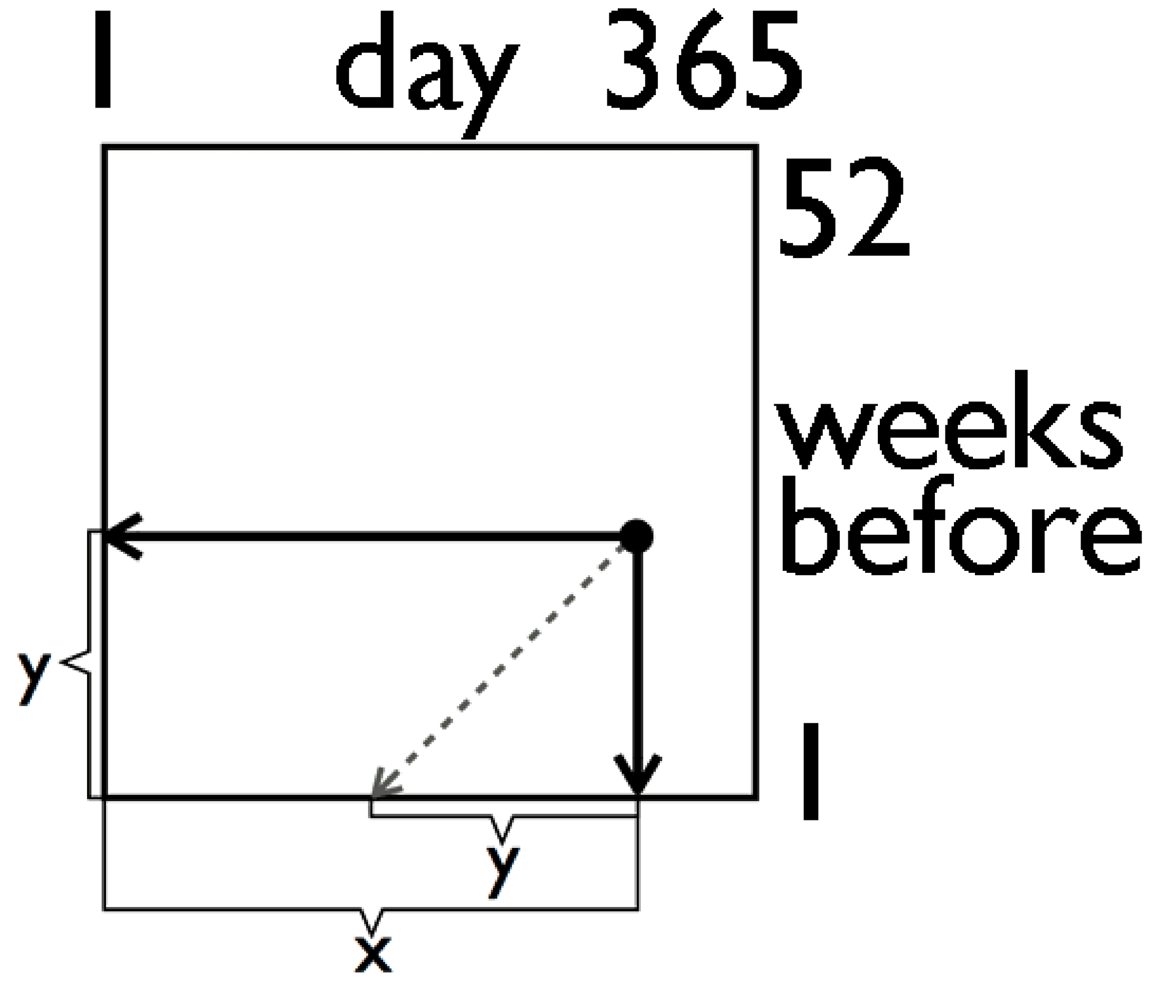
1
day
365
52
weeks
before
1
y
y
x

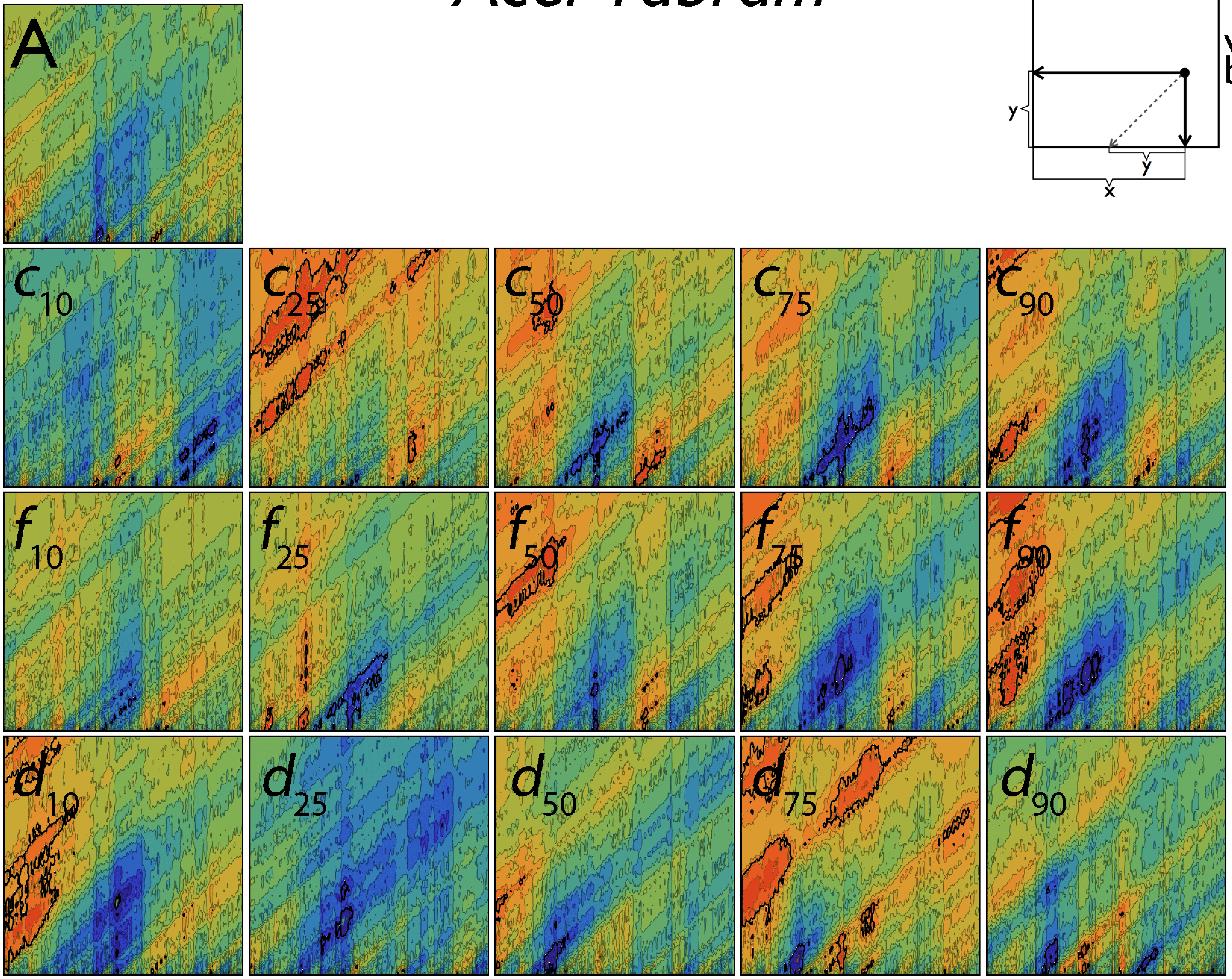
A
c10
c25
c50
c75
c90
f10
f25
f50
f75
f90
d10
d25
d50
d75
d90

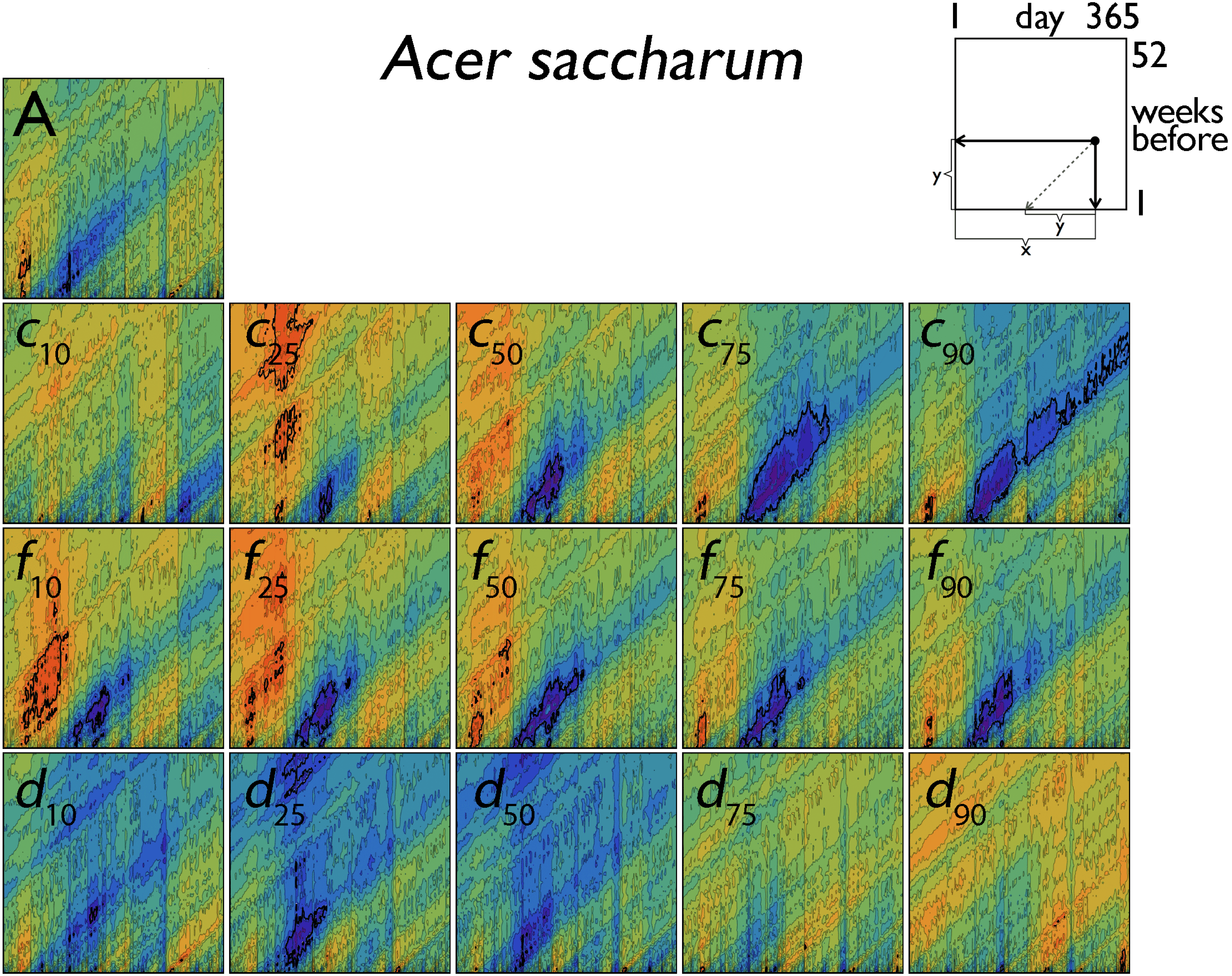
Acer saccharum
1
day
365
52
weeks before
1
y
y
x
A
c_{10}
c_{25}
c_{50}
c_{75}
c_{90}
f_{10}
f_{25}
f_{50}
f_{75}
f_{90}
d_{10}
d_{25}
d_{50}
d_{75}
d_{90}

Fraxinus americana

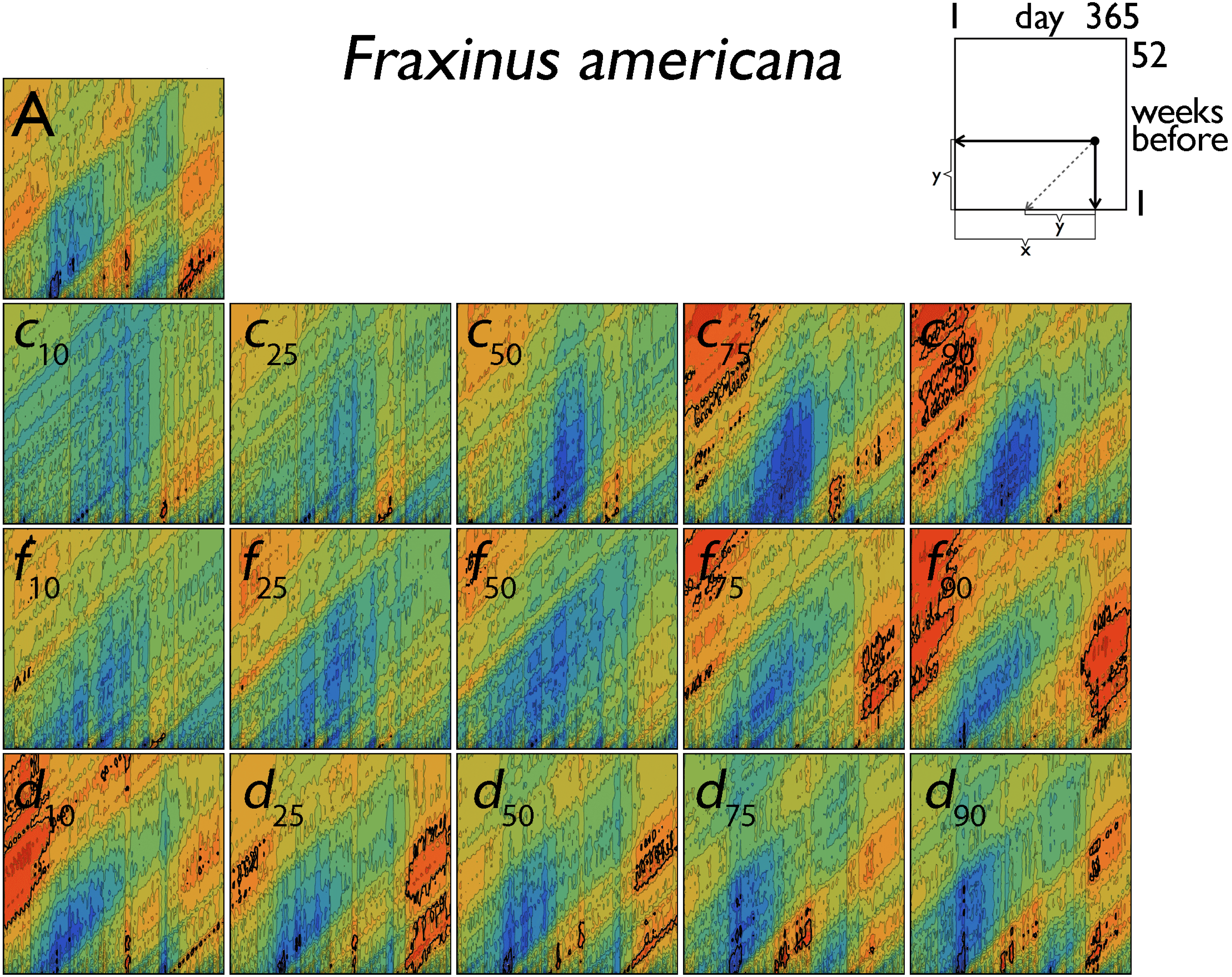

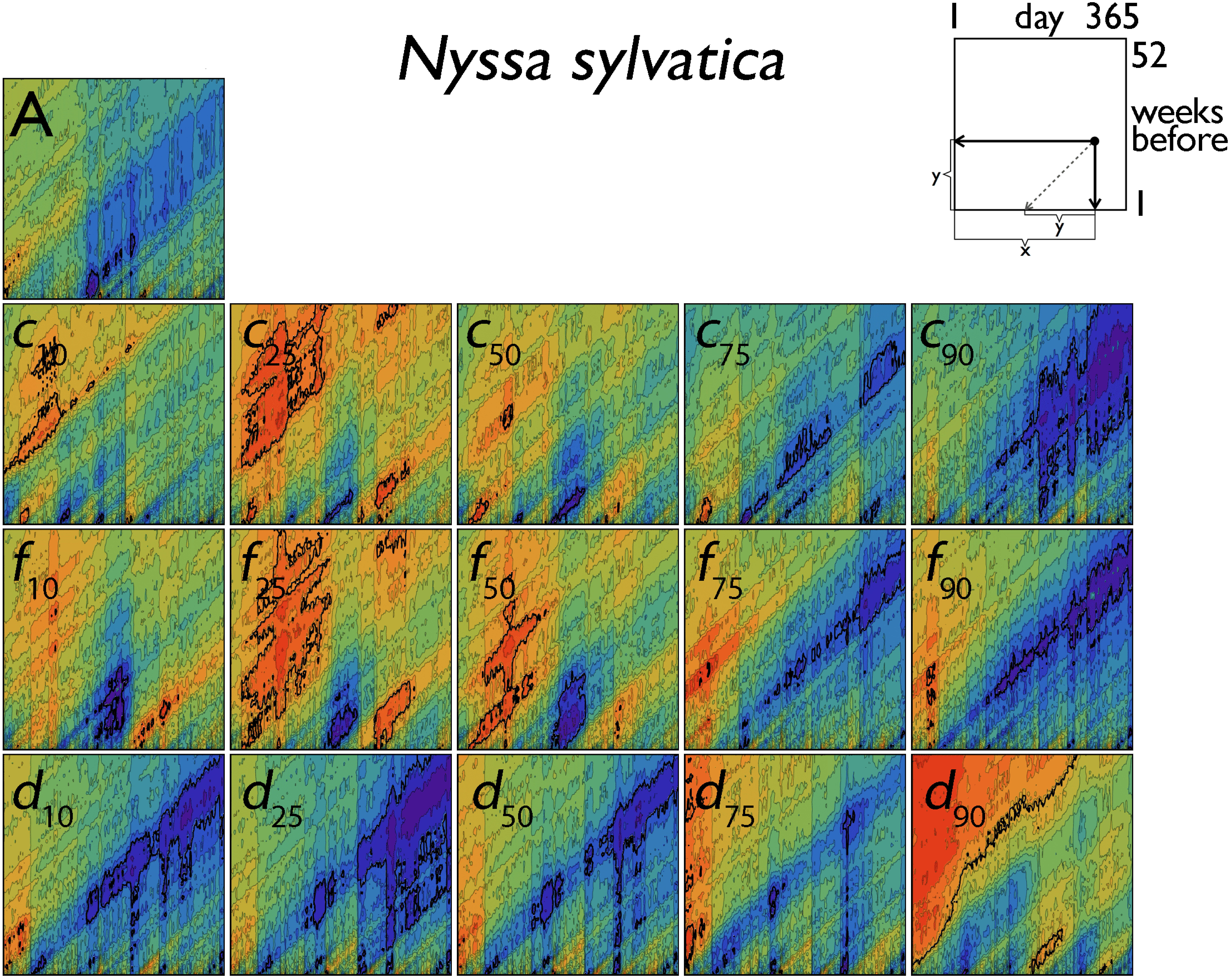

Nyssa sylvatica
1
day
365
52
weeks before
1
y
y
x
A
c_{10}
c_{25}
c_{50}
c_{75}
c_{90}
f_{10}
f_{25}
f_{50}
f_{75}
f_{90}
d_{10}
d_{25}
d_{50}
d_{75}
d_{90}

Prunus serotina

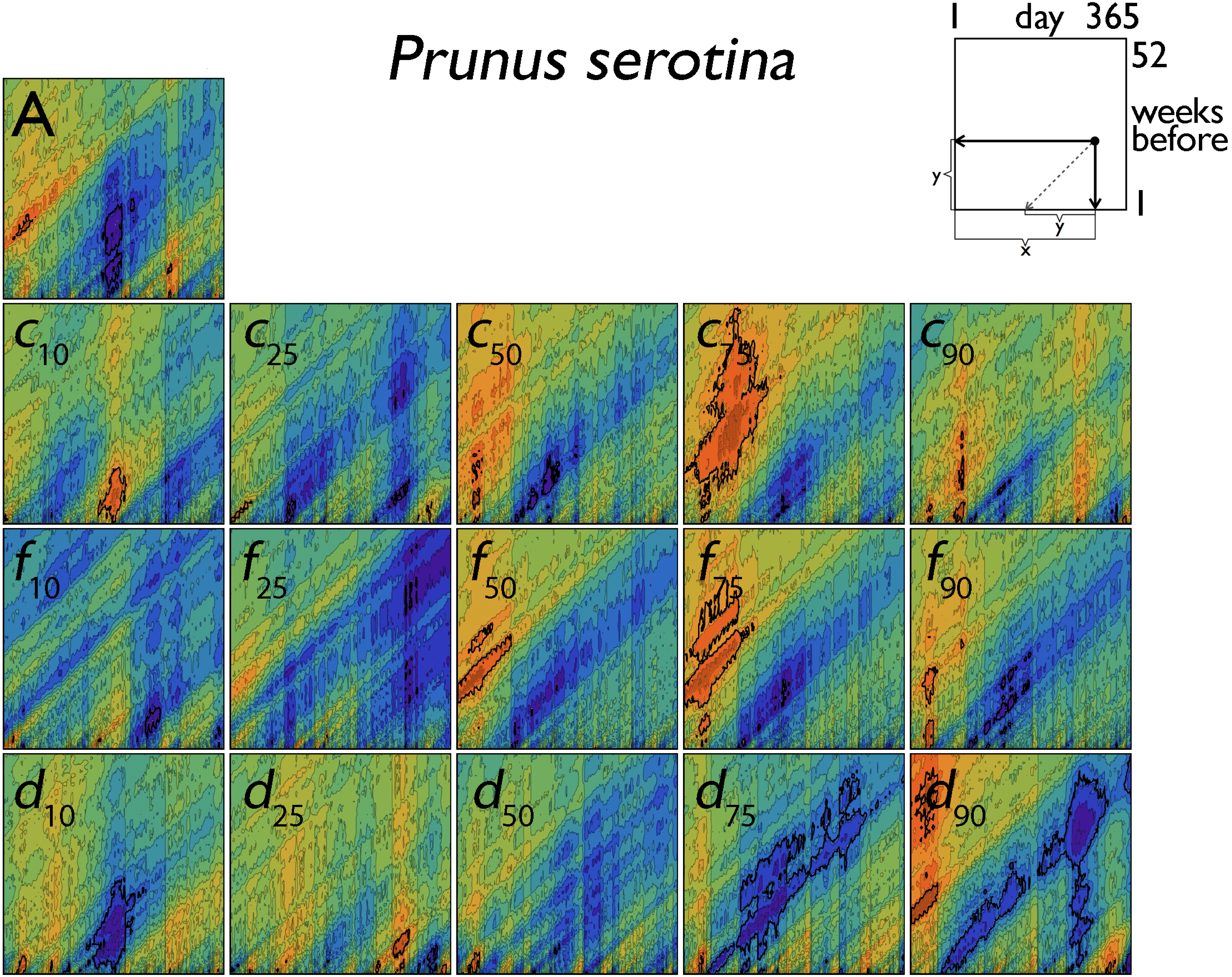

Quercus alba

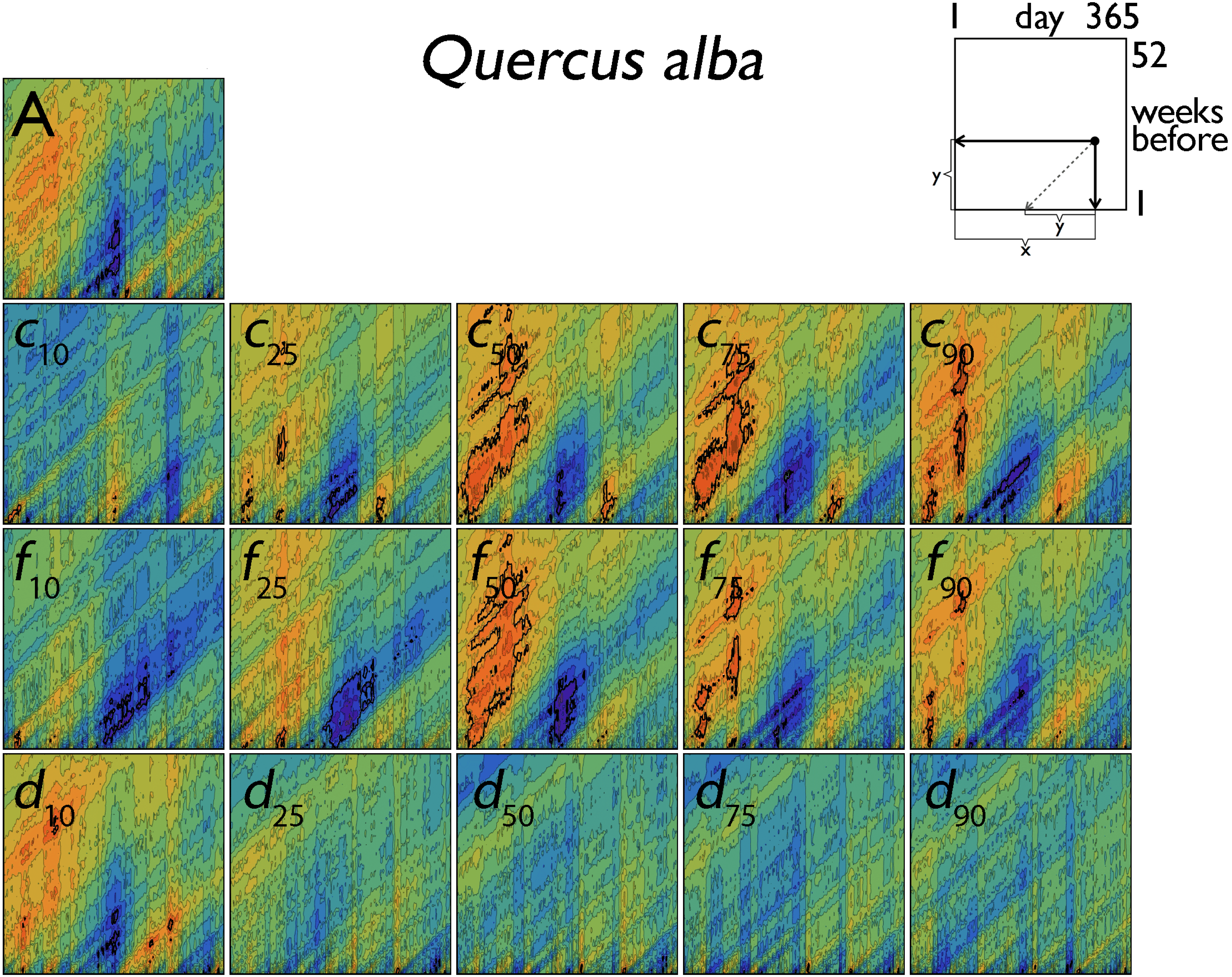

Quercus rubra

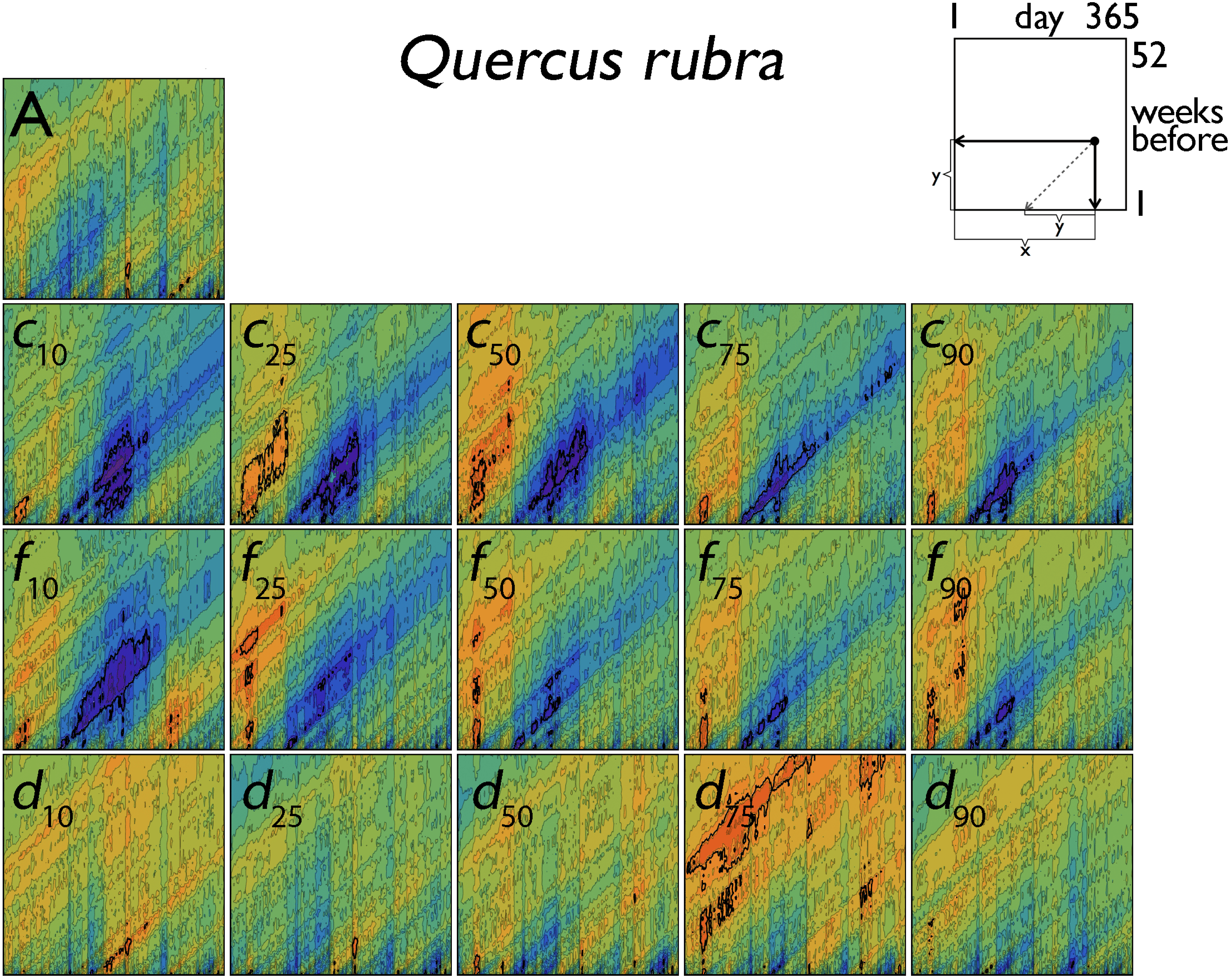

Quercus velutina

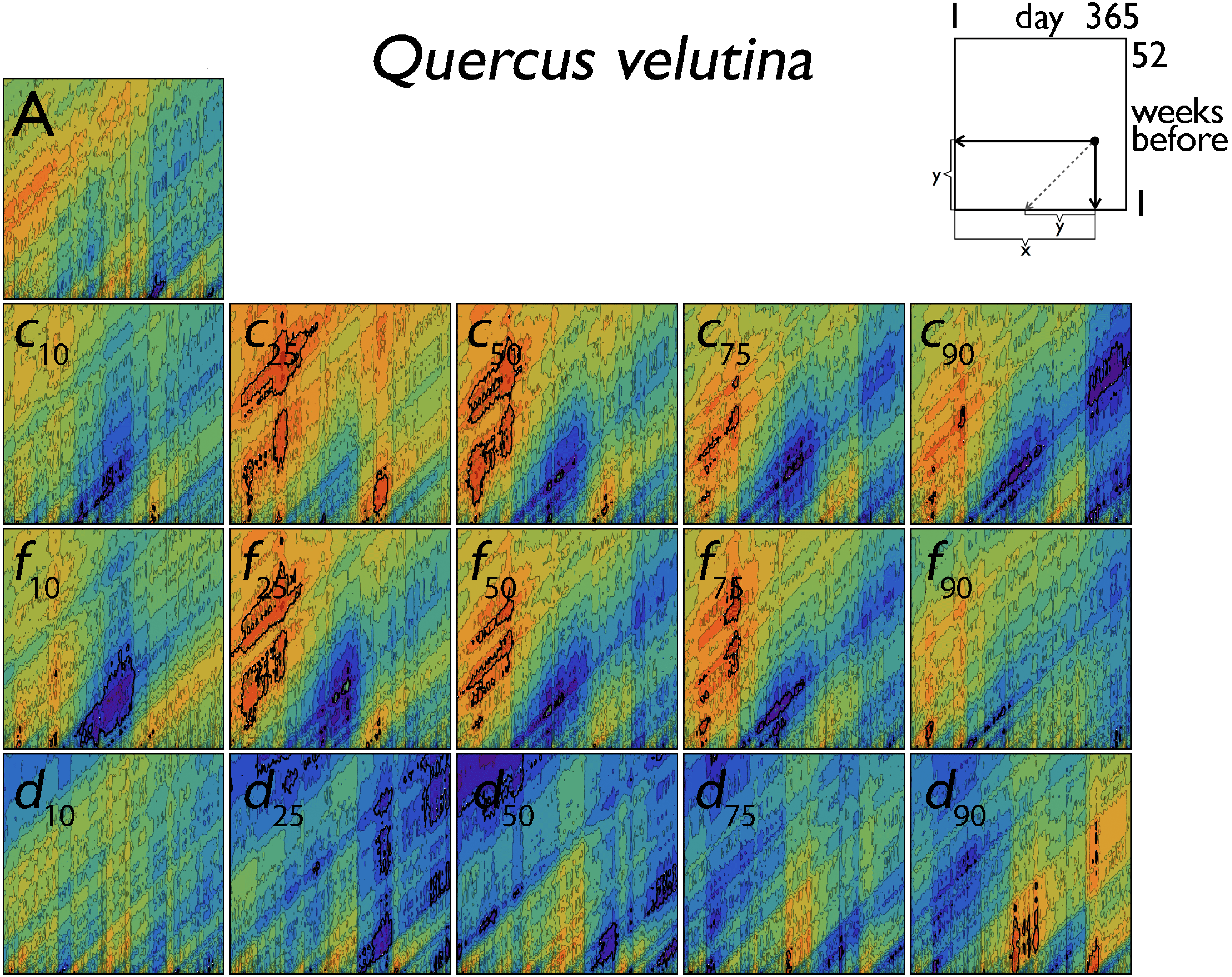

Supplement: Figure S2 — Impact of precipitation on the phenology of autumn colours and leaf fall. Same as Figure S1 but for precipitation rather than temperature. (PDF) [file pone.0057373.s002.pdf]
