# Supplementary material for: Predicting Climate Change Impacts on the Amount and Duration of Autumn Colors in a New England Forest
Source: PLoS One. 2013 Mar 8;8(3):e57373. doi: 10.1371/journal.pone.0057373 (PMC3592872; doi:10.1371/journal.pone.0057373)
Supplement: Table S2 — Parameters and statistics of model fits. Models were fit on the complete dataset. MLR model: P = number of parameters estimated in regression model. Temperature and Precipitation columns indicate months that were selected for inclusion in the regression model. + and − signs denote the sign of the regression coefficient. CDD/P model: parameters described in the text. F(P(doy)) refers to the use of the first or second function for simulating the interacting effect of photoperiod on the temperature dependence of phenological processes (see text for details). (DOC) [file pone.0057373.s004.doc]

**Parameters and statistics of model fits.** Models were fit on the complete dataset. MLR model: P = number of parameters estimated in regression model. Temperature and Precipitation columns indicate months that were selected for inclusion in the regression model. + and – signs denote the sign of the regression coefficient. CDD/P model: parameters described in the text. F(P(*d*)) refers to the use of the first or second function for simulating the interacting effect of photoperiod on the temperature dependence of phenological processes (see text for details). ACRU: *Acer rubrum*; ACSA: *Acer saccharum*; FRAM: *Fraxinus americana*; NYSY: *Nyssa sylvatica*; PRSE: *Prunus serotina*; QUAL: *Quercus alba*; QURU: *Quercus rubra*: QUVE: *Quercus velutina.*

|  |  | **MLR model** | | | | | | | | | | | | | | | **CDD/P model** | | | | | | | |
| --- | --- | --- | --- | --- | --- | --- | --- | --- | --- | --- | --- | --- | --- | --- | --- | --- | --- | --- | --- | --- | --- | --- | --- | --- |
| Stage | Species | Temperature | | | | | | Precipitation | | | | | |  |  |  |  |  |  |  |  |  |  |  |
|  |  | May | Jun | Jul | Aug | Sep | Oct | May | Jun | Jul | Aug | Sep | Oct | P | RMSE | R² | Pstart | Tb | f(P(doy)) | x | y | Ycrit | RMSE | R² |
| c_10 | ACRU |  |  |  |  |  |  |  |  |  |  |  |  | 1 | 3.18 | 0 | 13 | 30 | 1 | 1 | 2 | 107 | 2.97 | 0.09 |
| c_25 | ACRU |  |  |  |  |  |  |  |  |  |  |  |  | 1 | 3.76 | 0 | 13 | 30 | 1 | 1 | 2 | 186 | 2.47 | 0.59 |
| c_50 | ACRU |  |  |  |  | **+** |  |  |  |  |  |  |  | 2 | 2.4 | 0.4 | 13 | 30 | 1 | 1 | 2 | 254 | 2.27 | 0.44 |
| c_75 | ACRU | **-** |  |  |  | **+** |  |  |  |  |  |  |  | 3 | 1.93 | 0.72 | 13 | 30 | 1 | 1 | 2 | 316 | 1.90 | 0.68 |
| c_90 | ACRU |  |  |  |  | **+** | **+** |  |  |  |  |  |  | 3 | 1.99 | 0.75 | 13 | 30 | 1 | 1 | 2 | 359 | 1.68 | 0.80 |
| c_10 | ACSA |  |  |  | **+** |  |  |  |  |  |  |  |  | 2 | 5.6 | 0.25 | 14 | 28 | 2 | 1 | 1 | 71 | 5.08 | 0.40 |
| c_25 | ACSA | **-** |  |  |  | **+** |  |  |  |  |  |  |  | 3 | 3.31 | 0.5 | 14 | 28 | 2 | 1 | 1 | 114 | 3.70 | 0.27 |
| c_50 | ACSA | **-** |  |  |  | **+** | **+** |  |  |  |  |  |  | 4 | 2.04 | 0.7 | 14 | 28 | 2 | 1 | 1 | 146 | 2.25 | 0.54 |
| c_75 | ACSA |  |  |  |  | **+** |  |  |  | **+** |  | **-** |  | 4 | 2 | 0.66 | 14 | 28 | 2 | 1 | 1 | 174 | 2.11 | 0.56 |
| c_90 | ACSA |  |  |  |  |  |  |  |  |  |  |  |  | 1 | 3.45 | 0 | 14 | 28 | 2 | 1 | 1 | 194 | 3.05 | 0.24 |
| c_10 | FRAM | **-** |  |  |  |  |  |  |  |  |  |  |  | 2 | 3.59 | 0.43 | 14 | 30 | 1 | 1 | 0 | 429 | 4.47 | 0.06 |
| c_25 | FRAM | **-** |  |  |  |  | **-** |  |  |  | **-** |  |  | 4 | 2.23 | 0.78 | 14 | 30 | 1 | 1 | 0 | 525 | 3.24 | 0.39 |
| c_50 | FRAM | **-** |  |  | **+** |  |  |  |  |  |  |  |  | 3 | 3.07 | 0.63 | 14 | 30 | 1 | 1 | 0 | 626 | 4.22 | 0.21 |
| c_75 | FRAM |  |  | **-** |  | **+** |  |  |  |  |  |  |  | 3 | 3.37 | 0.61 | 14 | 30 | 1 | 1 | 0 | 821 | 4.26 | 0.26 |
| c_90 | FRAM | **-** |  | **-** |  | **+** |  |  |  |  |  |  |  | 4 | 3.29 | 0.67 | 14 | 30 | 1 | 1 | 0 | 941 | 3.82 | 0.44 |
| c_10 | NYSY |  |  |  |  |  |  |  |  |  |  |  |  | 1 | 3.38 | 0 | 14.5 | 28 | 2 | 1 | 1 | 69 | 2.98 | 0.21 |
| c_25 | NYSY |  |  |  | **+** |  |  |  |  |  |  |  |  | 2 | 3.02 | 0.52 | 14.5 | 28 | 2 | 1 | 1 | 83 | 3.14 | 0.43 |
| c_50 | NYSY |  |  |  | **+** |  |  |  |  |  |  |  |  | 2 | 2.8 | 0.56 | 14.5 | 28 | 2 | 1 | 1 | 101 | 3.70 | 0.20 |
| c_75 | NYSY |  |  |  | **+** |  |  |  |  |  |  |  |  | 2 | 4.45 | 0.29 | 14.5 | 28 | 2 | 1 | 1 | 126 | 4.20 | 0.28 |
| c_90 | NYSY |  |  |  |  |  |  |  |  |  |  |  |  | 1 | 6.8 | 0 | 14.5 | 28 | 2 | 1 | 1 | 180 | 5.54 | 0.36 |
| c_10 | PRSE |  |  |  |  |  | **-** | **-** |  |  |  |  |  | 3 | 4.5 | 0.59 | 14 | 26 | 2 | 1 | 1 | 38 | 5.21 | 0.37 |
| c_25 | PRSE |  | **+** |  |  |  |  |  |  |  |  |  |  | 2 | 6.26 | 0.43 | 14 | 26 | 2 | 1 | 1 | 79 | 7.18 | 0.20 |
| c_50 | PRSE |  |  |  |  | **+** |  |  |  |  |  |  |  | 2 | 3.11 | 0.41 | 14 | 26 | 2 | 1 | 1 | 121 | 2.45 | 0.60 |
| c_75 | PRSE |  |  |  |  | **+** |  |  |  |  |  | **-** |  | 3 | 3.06 | 0.49 | 14 | 26 | 2 | 1 | 1 | 150 | 2.76 | 0.50 |
| c_90 | PRSE |  |  |  |  |  |  |  |  |  |  |  |  | 1 | 5.44 | 0 | 14 | 26 | 2 | 1 | 1 | 190 | 4.56 | 0.26 |
| c_10 | QUAL |  |  |  |  |  |  |  |  |  |  |  |  | 1 | 3.24 | 0 | 14 | 30 | 1 | 1 | 2 | 342 | 3.92 | 0.01 |
| c_25 | QUAL | **-** |  | **-** |  | **+** |  |  |  |  |  |  |  | 4 | 2.87 | 0.74 | 14 | 30 | 1 | 1 | 2 | 428 | 3.80 | 0.41 |
| c_50 | QUAL |  |  | **-** |  | **+** |  |  |  |  |  |  |  | 3 | 2.02 | 0.86 | 14 | 30 | 1 | 1 | 2 | 534 | 2.71 | 0.71 |
| c_75 | QUAL |  |  | **-** |  | **+** |  |  |  |  |  |  | **+** | 4 | 2.22 | 0.83 | 14 | 30 | 1 | 1 | 2 | 598 | 2.37 | 0.75 |
| c_90 | QUAL | **-** | **-** |  |  | **+** |  |  |  |  |  |  |  | 4 | 2.46 | 0.83 | 14 | 30 | 1 | 1 | 2 | 657 | 3.49 | 0.57 |
| c_10 | QURU | **-** |  |  | **+** |  |  |  |  |  |  |  |  | 3 | 3.43 | 0.57 | 14 | 30 | 1 | 1 | 1 | 543 | 3.99 | 0.32 |
| c_25 | QURU |  |  | **-** |  | **+** |  |  |  |  |  |  |  | 3 | 2.52 | 0.65 | 14 | 30 | 1 | 1 | 1 | 659 | 2.40 | 0.63 |
| c_50 | QURU |  |  |  |  | **+** |  |  |  | **+** |  | **-** |  | 4 | 1.8 | 0.77 | 14 | 30 | 1 | 1 | 1 | 754 | 1.90 | 0.71 |
| c_75 | QURU |  |  |  |  | **+** |  |  |  |  |  |  |  | 2 | 3.42 | 0.3 | 14 | 30 | 1 | 1 | 1 | 853 | 3.13 | 0.40 |
| c_90 | QURU |  |  |  |  | **+** |  |  |  |  |  |  |  | 2 | 4.16 | 0.29 | 14 | 30 | 1 | 1 | 1 | 923 | 3.75 | 0.36 |
| c_10 | QUVE | **-** |  | **-** |  | **+** | **-** |  |  |  |  |  |  | 5 | 1.84 | 0.82 | 14 | 29 | 1 | 1 | 2 | 359 | 2.78 | 0.48 |
| c_25 | QUVE |  |  | **-** |  | **+** |  |  |  |  |  |  |  | 3 | 2.59 | 0.7 | 14 | 29 | 1 | 1 | 2 | 440 | 2.99 | 0.55 |
| c_50 | QUVE |  |  | **-** |  | **+** |  |  |  |  |  |  |  | 3 | 2.16 | 0.81 | 14 | 29 | 1 | 1 | 2 | 505 | 2.07 | 0.79 |
| c_75 | QUVE |  |  |  |  | **+** |  |  |  |  |  |  | **+** | 3 | 3.3 | 0.66 | 14 | 29 | 1 | 1 | 2 | 556 | 2.91 | 0.68 |
| c_90 | QUVE |  |  |  |  | **+** |  |  |  |  |  |  | **+** | 3 | 3.79 | 0.65 | 14 | 29 | 1 | 1 | 2 | 605 | 4.20 | 0.49 |
| f_10 | ACRU | **-** |  | **-** |  | **+** |  |  |  | **-** |  |  |  | 5 | 1.82 | 0.81 | 13 | 30 | 1 | 1 | 1 | 301 | 3.05 | 0.26 |
| f_25 | ACRU | **-** |  |  |  | **+** |  |  |  |  |  |  |  | 3 | 2.39 | 0.53 | 13 | 30 | 1 | 1 | 1 | 378 | 2.72 | 0.33 |
| f_50 | ACRU | **-** |  |  |  | **+** |  |  |  |  |  |  |  | 3 | 1.68 | 0.75 | 13 | 30 | 1 | 1 | 1 | 458 | 2.01 | 0.61 |
| f_75 | ACRU |  |  |  |  | **+** |  |  |  |  |  |  |  | 2 | 1.76 | 0.71 | 13 | 30 | 1 | 1 | 1 | 518 | 1.90 | 0.66 |
| f_90 | ACRU |  |  |  |  | **+** |  |  |  |  |  |  |  | 2 | 2.09 | 0.61 | 13 | 30 | 1 | 1 | 1 | 553 | 1.93 | 0.64 |
| f_10 | ACSA |  |  |  |  | **+** |  |  |  |  |  |  |  | 2 | 4.45 | 0.28 | 14 | 30 | 1 | 1 | 0 | 841 | 3.22 | 0.61 |
| f_25 | ACSA |  |  |  |  | **+** |  |  |  |  |  |  |  | 2 | 3.11 | 0.43 | 14 | 30 | 1 | 1 | 0 | 979 | 2.22 | 0.68 |
| f_50 | ACSA |  |  |  |  | **+** |  |  |  |  |  |  |  | 2 | 3.17 | 0.42 | 14 | 30 | 1 | 1 | 0 | 1111 | 2.58 | 0.58 |
| f_75 | ACSA |  |  |  |  | **+** |  |  |  |  |  |  |  | 2 | 3.62 | 0.36 | 14 | 30 | 1 | 1 | 0 | 1205 | 2.90 | 0.55 |
| f_90 | ACSA |  |  |  |  | **+** | **+** |  |  |  |  |  |  | 3 | 3.42 | 0.52 | 14 | 30 | 1 | 1 | 0 | 1315 | 3.21 | 0.50 |
| f_10 | FRAM |  |  |  |  |  |  |  |  |  |  |  |  | 1 | 5.22 | 0 | 13 | 7 | 1 | 0 | 0 | 273 | 5.08 | 0 |
| f_25 | FRAM | **-** |  |  |  |  |  |  |  |  |  |  |  | 2 | 4.37 | 0.25 | 13 | 7 | 1 | 0 | 0 | 276 | 4.77 | 0 |
| f_50 | FRAM |  |  | **-** |  |  | **-** |  |  |  |  |  |  | 3 | 3.58 | 0.54 | 13 | 7 | 1 | 0 | 0 | 282 | 4.84 | 0 |
| f_75 | FRAM |  |  |  |  |  |  |  |  |  |  |  |  | 1 | 5.16 | 0 | 13 | 7 | 1 | 0 | 0 | 290 | 5.03 | 0 |
| f_90 | FRAM |  |  |  |  |  |  |  |  |  |  |  |  | 1 | 5.75 | 0 | 13 | 7 | 1 | 0 | 0 | 295 | 5.59 | 0 |
| f_10 | NYSY | **-** |  |  | **+** |  |  | **+** |  |  |  |  |  | 4 | 2.06 | 0.74 | 14 | 30 | 1 | 1 | 1 | 541 | 2.84 | 0.40 |
| f_25 | NYSY |  |  | **-** | **+** | **+** |  |  |  |  |  |  |  | 4 | 2.81 | 0.73 | 14 | 30 | 1 | 1 | 1 | 588 | 3.76 | 0.41 |
| f_50 | NYSY |  |  | **-** | **+** |  |  | **+** |  |  |  |  |  | 4 | 2.31 | 0.74 | 14 | 30 | 1 | 1 | 1 | 641 | 3.71 | 0.26 |
| f_75 | NYSY |  |  |  | **+** |  |  |  |  |  |  |  |  | 2 | 4.62 | 0.23 | 14 | 30 | 1 | 1 | 1 | 775 | 4.26 | 0.28 |
| f_90 | NYSY |  |  |  |  | **+** |  |  |  | **+** |  |  |  | 3 | 4.7 | 0.63 | 14 | 30 | 1 | 1 | 1 | 889 | 5.68 | 0.37 |
| f_10 | PRSE |  |  |  |  |  |  |  |  |  |  |  |  | 1 | 7.64 | 0 | 14 | 30 | 1 | 1 | 1 | 517 | 8.59 | 0.02 |
| f_25 | PRSE |  |  |  |  |  |  |  |  |  |  |  |  | 1 | 7.79 | 0 | 14 | 30 | 1 | 1 | 1 | 678 | 7.42 | 0.08 |
| f_50 | PRSE |  |  |  |  | **+** |  |  |  |  |  |  |  | 2 | 5.1 | 0.26 | 14 | 30 | 1 | 1 | 1 | 793 | 4.47 | 0.36 |
| f_75 | PRSE |  |  |  |  | **+** |  |  |  |  |  |  |  | 2 | 4.31 | 0.37 | 14 | 30 | 1 | 1 | 1 | 880 | 3.92 | 0.42 |
| f_90 | PRSE |  |  |  |  | **+** |  |  |  |  |  |  |  | 2 | 4.55 | 0.33 | 14 | 30 | 1 | 1 | 1 | 938 | 4.28 | 0.34 |
| f_10 | QUAL |  |  | **-** |  |  | **-** |  |  |  |  |  |  | 3 | 2.69 | 0.6 | 14 | 30 | 1 | 1 | 2 | 492 | 5.03 | 0.02 |
| f_25 | QUAL |  |  |  |  |  |  |  | **+** |  |  |  |  | 2 | 3.64 | 0.36 | 14 | 30 | 1 | 1 | 2 | 572 | 4.31 | 0.20 |
| f_50 | QUAL |  | **-** |  |  | **+** |  |  |  |  |  |  |  | 3 | 3.73 | 0.72 | 14 | 30 | 1 | 1 | 2 | 668 | 4.45 | 0.53 |
| f_75 | QUAL |  |  |  |  | **+** |  |  |  |  |  |  |  | 2 | 4.61 | 0.54 | 14 | 30 | 1 | 1 | 2 | 742 | 5.03 | 0.39 |
| f_90 | QUAL |  | **-** |  |  | **+** |  |  |  |  |  |  |  | 3 | 4.15 | 0.65 | 14 | 30 | 1 | 1 | 2 | 795 | 5.39 | 0.31 |
| f_10 | QURU |  |  |  |  |  |  |  |  |  |  |  |  | 1 | 4.05 | 0 | 14 | 29 | 1 | 1 | 1 | 737 | 3.74 | 0.30 |
| f_25 | QURU |  |  |  |  | **+** |  |  |  |  |  |  |  | 2 | 3.92 | 0.34 | 14 | 29 | 1 | 1 | 1 | 822 | 3.79 | 0.34 |
| f_50 | QURU |  |  |  |  | **+** |  |  |  |  |  |  |  | 2 | 4.31 | 0.4 | 14 | 29 | 1 | 1 | 1 | 916 | 4.37 | 0.32 |
| f_75 | QURU |  |  |  |  | **+** |  |  |  |  |  |  |  | 2 | 4.54 | 0.32 | 14 | 29 | 1 | 1 | 1 | 985 | 4.49 | 0.27 |
| f_90 | QURU |  |  |  |  | **+** |  |  |  |  |  |  |  | 2 | 4.83 | 0.35 | 14 | 29 | 1 | 1 | 1 | 1051 | 4.73 | 0.30 |
| f_10 | QUVE | **-** |  | **-** |  | **+** |  | **+** |  |  |  |  |  | 5 | 1.06 | 0.94 | 14 | 30 | 1 | 1 | 2 | 510 | 3.39 | 0.35 |
| f_25 | QUVE |  |  |  |  | **+** |  |  |  |  |  | **-** |  | 3 | 2.48 | 0.79 | 14 | 30 | 1 | 1 | 2 | 585 | 2.35 | 0.79 |
| f_50 | QUVE |  |  |  |  | **+** |  |  |  |  |  |  |  | 2 | 3.49 | 0.56 | 14 | 30 | 1 | 1 | 2 | 647 | 3.04 | 0.63 |
| f_75 | QUVE |  |  |  |  | **+** |  |  |  |  |  |  |  | 2 | 3.88 | 0.5 | 14 | 30 | 1 | 1 | 2 | 704 | 3.79 | 0.47 |
| f_90 | QUVE |  |  |  |  | **+** |  |  |  |  |  |  |  | 2 | 4.28 | 0.29 | 14 | 30 | 1 | 1 | 2 | 764 | 4.73 | 0.18 |
